# Digital assets: risks, regulations, mitigation

**DOI:** 10.1186/s40854-025-00848-y

**Published:** 2026-02-09

**Authors:** Huei-Wen Teng, Wolfgang Karl Härdle, Joerg Osterrieder, Daniel Traian Pele, Lennart John Baals, Vassilios Papavassiliou, Karolina Bolesta, Audrius Kabašinskas, Olivija Filipovska, Nikolaos S. Thomaidis, Alexios-Ioannis Moukas, Sam Goundar, Jamal Abdul Nasir, Abraham Itzhak Weinberg, Veni Arakelian, Ciprian-Octavian Truică, Mutlu Akar, Esra Kabaklarlı, Elena-Simona Apostol, Maria Iannario, Barbara Bȩdowska-Sójka, Hanna Kristín Skaftadóttir, Ozgur Yildirim, Albulena Shala, Galena Pisoni, Ioana Florina Coita, Szabolcs Korba, Christian M. Hafner, Peter Schwendner, Bálint Molnár, Elda Xhumari

**Affiliations:** 1 Blockchain Research Center, Humboldt-Universität zu Berlin, Berlin, Germany; 2 Department Mathematics and Physics, Charles University, Prague, Czech Republic; 3 Sim Kee Boon Institute, Singapore Management U, Singapore, Singapore; 4 ACI Asia Competitiveness Institute, National University Singapore, Singapore, Singapore; 5 IDA Institute of Digital Assets, Bucharest University of Economic Studies, Bucharest, Romania; 6 Institute of Applied Data Science and Finance, Bern Business School, Brückenstrasse 73, Bern, Switzerland; 7 The High-Tech Business and Entrepreneurship Group, Faculty of Behavioural, Management and Social Sciences, University of Twente, Enschede, The Netherlands; 8 Institute for Economic Forecasting, Romanian Academy, Bucharest, Romania; 9 Bern University of Applied Science, Bern, Switzerland; 10 University College Dublin, UCD Michael Smurfit Graduate Business School and UCD Geary Institute for Public Policy, Carysfort Avenue, Blackrock, Co Dublin Ireland; 11 Department of Economics, Warsaw School of Economics, Warsaw, Poland; 12 Department of Mathematical Modeling, Kaunas University of Technology, Kaunas, Lithuania; 13 Komercijalna Banka AD Skopje, Skopje, North Macedonia; 14 Department of Financial and Management Engineering, University of the Aegean, Chios, Greece; 15 School of Economics, Aristotle University of Thessaloniki, Thessaloníki, Greece; 16 School of Science and Technology, RMIT University, Hanoi, Vietnam; 17 University of Galway, Galway, Ireland; 18 AI-Weinberg, Tel Aviv, Israel; 19 Economic Research and Investment Strategy, Piraeus Bank, Athens, Greece; 20 UCL Center for Blockchain Technologies, London, UK; 21 University Politehnica of Bucharest, Bucharest, Romania; 22 College of Arts and Sciences, Department of Mathematics, Yildiz Technical University, Istanbul, Türkiye; 23 Department of Economics, Seluk University, Konya, Turkey; 24 University of Naples Federico II, Naples, Italy; 25 Poznań University of Economics and Business, Poznan, Poland; 26 University of Iceland, Reykjavík, Iceland; 27 University of Prishtina “Hasan Prishtina”, Prishtina, Kosova; 28 Universite Cote d’Azur, Biot, France; 29 University of Oradea, Oradea, Romania; 30 Eötvös Loránd University, Budapest, Hungary; 31 Université catholique de Louvain, Louvain-la-Neuve, Belgium; 32 Institute of Wealth and Asset Management, Zurich University of Applied Sciences, Winterthur, Switzerland; 33 Tirana University, Tirana, Albania

**Keywords:** Digital asset, Blockchain, Regulatory framework, Decentralized finance, Non-fungible token, G2, E4, K2, L5, O3, O1

## Abstract

Digital assets (DAs) such as cryptocurrencies, tokenized securities, stablecoins, non-fungible tokens (NFTs), and central bank digital currencies, are transforming financial markets with new business models, investment opportunities, and transaction efficiencies. Underpinned by blockchain, distributed ledger technology, and smart contracts, digital innovations are reshaping the financial ecosystem. However, their rapid growth introduces substantial risks, including fraud, market manipulation, cybersecurity threats, and regulatory uncertainty. This position paper offers an interdisciplinary and empirically grounded analysis of the DA landscape. We define and classify major asset types, trace their evolution from speculative instruments to functional tools, and assess current adoption trends. Additional technological developments (e.g., decentralized finance and NFT expansion) are examined for their role in accelerating this transformation. We also analyze the global regulatory landscape, highlighting jurisdictional differences, classification challenges, and emerging governance frameworks. To address key risks, we derive mitigation strategies via quantitative analysis and case-based evidence. The risks include balancing innovation with investor protection through adaptive regulatory design, promoting cross-border regulatory harmonization to prevent arbitrage and fragmentation, and supporting experimentation through regulatory sandboxes and innovation hubs. By adopting a forward-looking, evidence-based, and collaborative regulatory approaches, stakeholders can harness the benefits of DAs while managing systemic risks and maintaining market integrity.

## Digital assets (DAs)

The global proliferation of DAs in financial ecosystems has garnered significant attention from scholars, regulators, and market participants (Kuznetsov et al. 2020). These novel instruments pose unique opportunities and challenges, requiring a comprehensive understanding of their implications for financial stability and regulatory oversight. Limited interconnections with traditional financial systems and the lack of significant financial services outside digital DA paradigm renders it opaque and potentially fragile. This section first outlines the common DA terms and classifications to lay the groundwork for our in-depth analysis.

The evolution of DAs from novelty to necessity has been characterized by technological advancements, diversification, integration into the traditional financial system, and increasing institutional adoption. This trajectory elevates the importance of understanding and addressing the risks associated with their proliferation in order to maintain a stable and resilient global financial ecosystem.

Our paper makes important contributions by analyzing the risks associated with DAs and providing a detailed regulatory framework to mitigate these risks. Specifically, we propose an approach to handle emerging risks such as cybersecurity threats, environmental concerns, and socioeconomic inequalities. In addition, we offer a comprehensive examination of current DA ecosystems, integrating technological, regulatory, and economic perspectives to guide policymakers and investors in managing DA risks more effectively. We also highlight future developments in DAs, like NFTs and decentralized finance (DeFi), providing practical regulatory strategies. This contribution is essential for improving financial stability and ensuring inclusive DA ecosystems. Appendix A provides a summary of the abbreviations used in this paper.

A clear understanding of this taxonomy is essential for effective regulatory design and market compliance. Each DA category falls within a *distinct legal perimeter* (e.g., payment-instrument, securities, commodities, e-money, or sovereign-money law). This legal classification determines *which* authority is responsible for licensing the issuer, *which* prudential and disclosure requirements are applicable, and *which* consumer protection mechanisms are activated. Table 1 provides a summary of these regulatory linkages. Sections 2–4 build upon this framework to demonstrate how seemingly minor definitional changes can reclassify a token under an entirely different regulatory regime.

**Table 1:** Regulatory snap-shot by asset type

Asset class	Primary legal hook	Key obligations
Cryptocurrencies	Commodity/payment laws	AML/KYC, derivatives rules
Utility tokens	Often unregulated*	White-paper & travel-rule compliance
Security tokens	Securities law	Prospectus, custody, ongoing reporting
E-money stablecoins	E-money rules	1:1 reserves, attestations
Asset-referenced stbl.	“Significant ART” (MiCA)	Capital add-on, issuance caps
CBDCs	Central-bank statute	Privacy tiers, access limits
NFTs	Mostly $\mathrm{unregulated}^{\dag }$	IP safeguards, platform AML

* If marketed for investment, they may qualify as securities

$^{\dag }$ Fractional NFTs can likewise fall under securities law

### DA taxonomy

DAs, in the context of contemporary financial systems and technology, can be comprehensively defined as electronic data or content that possesses inherent, transactional, or functional value. See Toygar et al. (2013) for the seminal description. A DA is the digital representation of such value, cryptographically recorded on a secured DLT or comparable technology. The emergence and proliferation of these assets are largely driven by the ongoing digital transformation of the global economy, which has given rise to innovative business models, new investment opportunities, and evolving sources of risk.

These assets encompass a wide range of digital entities with diverse attributes and can be represented across multiple formats. DAs are created, stored, managed, and transacted through digital channels and technological infrastructures. At the core of this ecosystem is the blockchain—a DLT that, through decentralized applications (dApps), records transactions among network participants. Given their intrinsic complexity and rapidly evolving nature, DAs require careful and sustained analysis to fully understand their underlying features and their implications for both conventional and emerging financial systems. Such understanding is essential for designing strategies and regulatory frameworks that effectively mitigate associated risks while harnessing the opportunities offered by DA innovation in the financial sector.

Different regulatory bodies have proposed varying definitions and classification schemes for DAs. To develop a comprehensive understanding of DAs and their associated risks, it is essential to classify them according to their defining characteristics, functional roles, and intended use cases. In this study, we focus specifically on the most liquid categories of DAs. The following classification framework provides a practical basis for organizing and analyzing DAs.

- CCs: Decentralized digital currencies that utilize cryptographic techniques to secure transactions and control the creation of new blocks. Examples include Bitcoin (BTC), Ethereum (ETH), and Litecoin (LTC). They primarily function as a medium of exchange, a store of value, and a reward mechanism for maintaining blockchain networks (see, for example, Rowland and Kiviat 2018; Gencer et al. 2018; Rrustemi and Tuchschmid 2021).
- Utility tokens: DAs that grant access to specific products or services within a digital platform or ecosystem. They may serve various functions, such as enabling purchases, facilitating governance through voting, or unlocking exclusive content (Sonnino et al. 2019). Examples include the Basic Attention Token and Filecoin (FIL).
- Security Tokens: Digital representations of traditional financial securities, such as stocks, bonds, or real estate. These tokens are regulated under securities laws and confer rights related to ownership, dividends, profit sharing, or interest payments (see Farber et al. 2019; Lambert et al. 2020). Examples include tokenized equity shares and real estate investment tokens.
- Non-Fungible Tokens (NFTs): Unique DAs that signify ownership of specific assets or content, such as digital artwork, collectibles, or virtual real estate (Nadini et al. 2021). Each NFT has a distinct value recorded in a blockchain and is not interchangeable on a one-to-one basis with other NFTs. Representative examples include CryptoKitties and NBA Top Shot collectibles.
- Central Bank Digital Currencies (CBDCs): Digital fiat currencies (i.e., digital forms of sovereign money) issued by national central banks. Like traditional currencies, CBDCs serve as a medium of exchange, a store of value, and a unit of account (see Barrdear and Kumhof 2021; Bordo and Levin 2017). Notable examples include the e-CNY (Digital Yuan) and the proposed Digital Euro.
- Stablecoins: DAs designed to maintain a stable value by pegging their worth to a reserve of assets, such as fiat currencies, commodities, financial instruments, or other CCs. Prominent examples include Tether, USD Coin, and Digital Art Index (DAI) (Lin et al. 2022).

The classification of DAs is not mutually exclusive, as certain assets may exhibit characteristics that span multiple categories. Understanding the interactions and interdependencies among various types of DAs is critical, as these relationships can influence the overall risk profile of the ecosystem. For example, DeFi platforms often integrate a combination of CCs, utility tokens, and stablecoins to support lending, borrowing, and trading services. A meaningful analysis of these interconnections requires a dynamic network perspective that captures the variations in nodes and the degree of interconnectedness. An early example of such a network-based approach to CCs can be found in Guo et al. (2024) https://quantinar.com/course/50/cryptonetworks.

A recent synthesis paper by the International Monetary Fund (Financial Stability Board and International Monetary Fund 2023) summarized the primary risks associated with DAs from a macroeconomic stability perspective, concluding that widespread adoption could disrupt both monetary policy implementation and fiscal balance.

The six-group classification of DAs presented in this study naturally involves overlapping dimensions of technology and law. It can be further refined through the “landmine” framework proposed by Reyes (2024), which delineates nuanced distinctions among commonly used terms in blockchain and NFT discourse. That study emphasizes the need for researchers, lawmakers, industry participants, and other stakeholders to bridge conceptual gaps and ensure terminological precision. In the sections that follow, we provide additional discussion of terminology. The six categories outlined above represent a practical, hands-on taxonomy based on widely accepted DA terminology. Further clarification can be found in “landmine 3,” which critiques the confusion and misinterpretation that arise when terms such as cryptoassets, cryptocurrency (CC), DAs, and virtual currency are used interchangeably within legal frameworks and regulatory guidance.

### DA evolution

The emergence of DAs can be traced back to the early development of electronic money and digital cash systems. Although blockchain-based (BC) technology continues to lag behind credit card networks in terms of transaction speed, its adoption is accelerating rapidly and holds significant promise for future advancements. These early innovations laid the foundation for the DA ecosystem as it exists today.

Prior to the advent of BC CCs, multiple attempts were made to create digital currencies through centralized architectures. In the 1980s, David Chaum, a pioneer in cryptography and privacy, introduced the concept of eCash (Judmayer et al. 2022). Despite its technological innovation, eCash encountered numerous challenges, including limited user adoption, and its parent company, DigiCash, ultimately declared bankruptcy in 1998.

Subsequent efforts to establish centralized digital currencies included e-gold and Liberty Reserve. Launched in 1996, e-gold allowed users to store and transact digital representations of gold but later faced severe legal challenges related to money laundering, resulting in its closure in 2009. Similarly, Liberty Reserve, established in 2006, operated its own digital currency and facilitated online transfers. However, due to allegations of facilitating money laundering, the platform was shut down in 2013 (Dowd 2014). As illustrated in Table 2, the evolution of DAs encompasses several stages.

**Table 2:** DA evolution: from novelty to necessity

Stage	Description
Historical Context and Early DAs	The Emergence of digital currencies and early DA ecosystems
Blockchain Technology and CCs	Advent of blockchain technology, Bitcoin, and expansion of the CC landscape
Developments in Tokenization	Rise of initial coin offerings, token sales, and the tokenization of real-world assets
DeFi	The growth of DeFi platforms, services, and ecosystems
NFTs	The emergence of NFTs, digital collectibles, and their applications in various industries
CBDCs	Motivations, objectives, and developments in CBDC projects worldwide
Challenges and Risks	Security, infrastructure, regulatory, compliance, and environmental considerations in the evolution of DAs

These early DA ecosystems were constrained by several shortcomings, including centralized control, regulatory hurdles, and significant security vulnerabilities (Härdle et al. 2020). The emergence of BC technology and the introduction of BTC, a decentralized CC, helped overcome many of these limitations and represented a major milestone in the evolution of DAs.

### Advent of blockchain

Decentralization, enhanced security, and trustless transaction mechanisms are among the defining features that distinguish these novel assets from their predecessors. As an initial example, in 2008, an individual or group using the pseudonym Satoshi Nakamoto published the BTC whitepaper, which described the first implementation of a decentralized, peer-to-peer (P2P) electronic cash system (Nakamoto 2008). BTC’s underlying technology, the blockchain, is a DLT maintained by a network of nodes that employ cryptographic techniques to secure transactions and achieve consensus without the need for a central authority. BTC uses a proof-of-work (PoW) consensus mechanism, wherein miners (i.e., computers) verify transactions and append new blocks to the blockchain. This decentralized structure eliminated the need for intermediaries, such as banks or payment processors, thereby addressing many of the challenges that had hindered earlier digital currency systems.

**Fig. 1:**
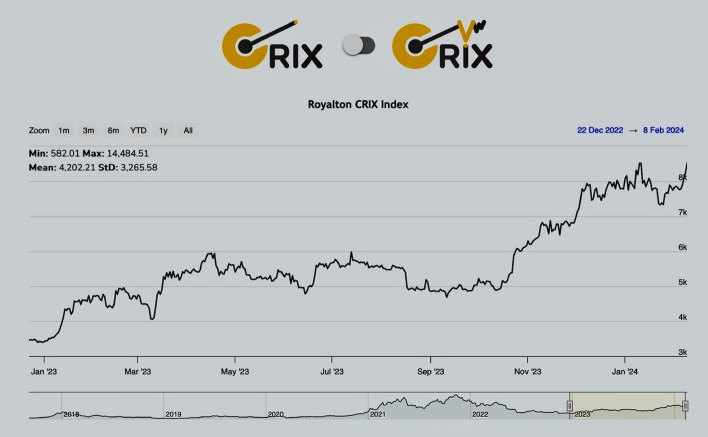
An adaptive CC index from Royalton Partners https://www.royalton-partners.com/royalton_crix_crypto_index/

Following BTC’s success, numerous alternative CCs (i.e., altcoins) emerged, many seeking to improve upon BTC’s design or target specialized use cases. ETH, launched in 2015, introduced a Turing-complete programming language and smart contract functionality, enabling developers to build dApps and automate complex transactions. LTC, another notable altcoin, was developed to offer faster transaction processing than BTC. The proliferation of these CCs fostered significant innovation in the DA space, giving rise to a diverse and expanding ecosystem comprising various blockchain platforms, token standards, and DA indices (Fig. 1).

The earliest known attempt to construct a DA index is, to the best of our knowledge, the CRypto currency IndeX (CRIX) (Trimborn and Härdle 2015), which is also listed by S&P Global. As of today, a general introduction to CCs is available in the courselet https://quantinar.com/course/22/crypto.

### NFTs

NFTs represent a distinct class of DAs that possess unique characteristics, in contrast to CCs, which are fungible and interchangeable. Built on BC technology—most commonly the ETH network—NFTs utilize token standards such as ERC-721 and ERC-1155 to create provably scarce, indivisible, and non-interchangeable digital tokens. In recent years, NFTs have gained substantial traction, particularly in the domains of digital art, collectibles, and virtual goods. Frye (2024) offered a concise history of NFTs and the broader NFT market, beginning with the invention of blockchain technology and tracing developments through the creation of BTC, Namecoin, and ETH, culminating in the rise of the NFT phenomenon. The study discusses key NFT projects and influential artists, while also providing a theoretical framework for understanding both the traditional art market and the evolving NFT market.

Indeed, NFTs emerged as an innovative means of tokenizing unique DAs, with their primary contribution being the creation of digital scarcity and the ability to establish verifiable ownership. NFTs can represent a wide spectrum of digital and physical assets, including artwork, virtual real estate, in-game items, domain names, and intellectual property.

Although NFTs are unique assets capable of generating economic value, they remain difficult to regulate. The concept of using tokens to represent rights and facilitate ownership transfer has historical precedents, evolving from physical instruments (e.g., paper certificates) to digital records. Negotiable instruments and certificated securities are early examples of tokenized obligations that became integrated into established commercial law; NFTs extend these traditions through the application of blockchain technology. Their use across diverse markets (e.g., art, collectibles, securities, and commodities) complicates efforts to develop a unified regulatory framework. Jurisdictions continue to diverge in their treatment of NFTs: for instance, U.S. congressional proposals often exclude NFTs or call for further examination, whereas the European Union’s cryptoassets framework largely omits them. At present, the regulatory environment for NFTs remains fragmented and subject to ongoing development.

The market for NFTs has experienced both exponential growth and sharp declines in recent years, driven by a range of applications and use cases. NFTs have disrupted the digital art and collectibles markets by enabling artists to tokenize their creations and sell them through dedicated NFT marketplaces. They offer artists new revenue streams by allowing them to earn royalties from secondary market sales. NFTs also play a pivotal role in virtual worlds and metaverse platforms, such as Decentraland https://decentraland.org and The Sandbox https://www.sandbox.game/en/nft/, where users can buy, sell, and trade virtual land, buildings, and other DAs represented as NFTs. Additionally, NFTs facilitate the tokenization of in-game items (e.g., skins, weapons, and characters), enabling verifiable ownership and the transfer of assets across different games and platforms. Beyond gaming, NFTs hold significant potential in intellectual property management and licensing, allowing creators to tokenize and monetize rights to music, software, patents, and other digital products.

**Fig. 2:**
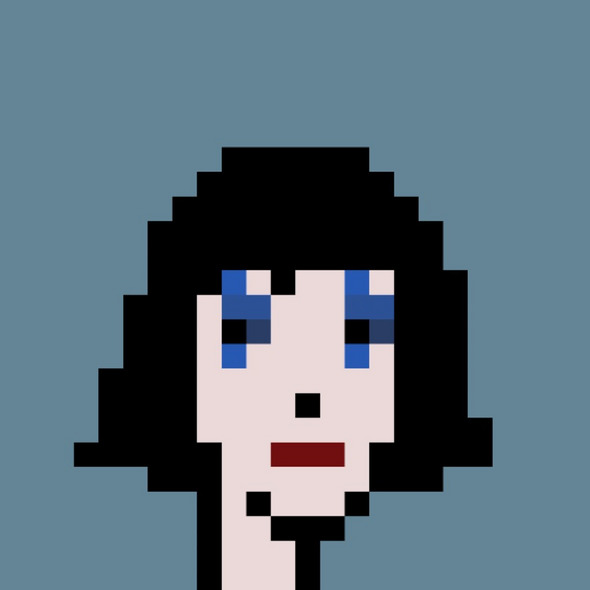
A cryptopunk from larvalabs.com

A prominent example of NFT-based business opportunities is illustrated by the extensive collection of digital artworks from larvalabs.com, including the well-known “CryptoPunks” series, a subset of NFTs showcased in Fig. 2.

NFTs have emerged as a nascent yet rapidly expanding phenomenon within the broader wave of digital transformation. In March 2021, an NFT (i.e., a single piece of digital art created by Mike Winklemann, also known as ‘Beeple”) sold for an unprecedented USD 69.3M. According to Grandviewresearch.com (January 2024), the global NFT market was valued at USD 26.9B in 2023 and DAs (tokens) is projected to grow at a compound annual growth rate of 34.5% from 2024 to 2030. NFTs take various forms, confer different rights, and are increasingly viewed as a significant asset class for investors. However, DAs, NFTs, and CCs remain highly volatile, with markets frequently experiencing sharp downturns and speculative price swings that can lead to substantial investor losses.

NFTs and DAs (tokens) are largely unregulated, and many central banks have so far adopted a “hands-off” approach. On the one hand, investments in these tokens continue to grow. On the other hand, the market remains highly volatile (Yousaf and Yarovaya 2022), posing substantial risks alongside potentially high returns. Investors, financial markets, and policymakers are therefore seeking clear guidelines and regulatory frameworks (Urom et al. 2022) to enable sustainable investment with minimized risk and optimized returns. Researchers such as Wilson et al. (2022) have examined NFTs across various industries, identifying both the opportunities they create and the risks they introduce. Moreover, Trevisi et al. (2022) discuss business models, legal aspects, and valuation approaches for NFTs, emphasizing that not all NFTs are created equal and that they confer differing rights. Consequently, legal frameworks and valuation methods require careful scrutiny to ensure that business models are constructed on sound foundations.

The NFT market is characterized by high risk and high return, generally operating with limited transparency and relatively low participation compared with conventional investment markets. Investors must therefore exercise caution and conduct thorough due diligence before entering the NFT space. The growing incidence of fraudulent activity and copyright infringement underscores the need for robust verification and validation mechanisms (Flick 2022).

### CBDCs and the future of money

CBDCs represent a novel form of digital money issued and backed by central banks. They have garnered significant attention from monetary authorities and policymakers worldwide, as jurisdictions explore both the potential advantages and challenges associated with implementing digital currencies within existing financial systems.

The design of a CBDC entails addressing several technical and economic considerations, including:

- Architecture: CBDCs can be structured using centralized, decentralized, or hybrid architectures, depending on the desired level of control and the roles assigned to the central bank and intermediary institutions (Zhang et al. 2021).
- Access: CBDCs may be designed for retail use (general public) or wholesale use (financial institutions), each with differing levels of accessibility and transactional functionality (Kochergin 2021).
- Privacy: Achieving an appropriate balance between privacy and regulatory compliance is a core design challenge, as central banks must safeguard user confidentiality while ensuring sufficient transparency to combat illicit activities such as money laundering and terrorist financing (Darbha and Arora 2020; Pocher and Veneris 2022).
- Interoperability: CBDCs should be designed to integrate seamlessly with existing payment infrastructures and other digital currencies, thereby facilitating efficient cross-border transactions and currency exchanges (Allen et al. 2020a).

Some countries, such as China and the Bahamas, have already launched pilot versions of their digital currencies. These initiatives can be broadly categorized into two main types:

- Retail CBDCs: Designed for use by the general public, retail CBDCs enable digital payments and transactions among consumers, businesses, and financial institutions. The Bahamian Sand Dollar and China’s Digital Currency Electronic Payment are examples of retail CBDCs currently in active circulation.
- Wholesale CBDCs: Targeted at financial institutions, wholesale CBDCs support interbank transactions and settlements, enhancing the efficiency and security of large-value financial operations. Illustrative initiatives include Project Ubin in Singapore https://www.mas.gov.sg/schemes-and-initiatives/project-ubin and Project Stella, a joint effort by the European Union and Japan https://www.ecb.europa.eu/paym/intro/publications/pdf/ecb.miptopical200212.en.pdf, both of which explore the potential applications and advantages of wholesale CBDCs.

Access to and circulation of CBDCs are typically restricted to designated agents operating under specific regulatory and policy frameworks, similar to the current management of central bank reserves. In principle, a wholesale CBDC represents an extension of existing reserve accounts held at the central bank, with electronic mobilization enabling eligible agents within a jurisdiction to access and utilize CBDC.

CBDC can be conceptualized as a third form of base money, complementing the existing overnight deposits with the central bank (currently accessible only to banks, select non-bank financial institutions, and some official-sector depositors) and banknotes (widely available but increasingly viewed as inefficient and reliant on outdated technology).

According to Bindseil (2019), two alternative technical formats could be employed to implement a general-purpose CBDC. First, households and corporations could be provided CBDC through deposit accounts at the central bank. This approach does not entail significant technological innovation but rather involves streamlining the number of existing bank accounts. Commercial banks would facilitate access to these accounts, charging a competitive service fee (comparable to current ATM fees) for converting traditional bank deposits into CBDC and banknotes. Second, the central bank could issue a digital token currency, operating without a central ledger and in a decentralized manner. This format is often associated with anonymity, implying that the central bank would be unable to track ownership of the issued tokens—analogous to the anonymity of physical banknotes.

World Bank (2021) outlined several key design features that should be considered in the development of a CBDC: anonymity, availability, interest-bearing capabilities, and the transfer mechanism (e.g., P2P transactions vs. intermediary-based transfers).

Studies by Fernández-Villaverde et al. (2021) and Schilling et al. (2024) examined the implications of CBDC in the context of bank runs. They found that the introduction of CBDC could reduce the risk of bank runs by incentivizing a migration of deposits from commercial banks to the central bank. Keister and Monnet (2022) further explored CBDC’s role in mitigating bank run risks, focusing on the efficacy of government interventions. Depending on its structural design, CBDC may enable central banks to more accurately assess the stability of the banking sector and respond promptly to emerging run scenarios.

Implementing CBDCs poses considerable challenges for central banks, encompassing political, economic, technical, and legal dimensions. A central question is whether households should be granted direct access to central bank balance sheets. Although CBDCs offer the potential to enhance the efficiency of financial services, technical complexities and political sensitivities raise doubts about their overall viability. Moreover, CBDCs may not guarantee financial stability or effective inflation management, as real-world dynamics could yield unpredictable outcomes. The implications for global currency holdings and the use of CBDCs in cross-border payments may introduce intricate coordination issues. Additionally, the blurred distinction between fiscal and monetary policy responsibilities introduced by CBDCs may not be well received by central banks (Chen and Siklos 2022).

From a payments perspective, the decision to adopt a CBDC should begin with a clear evaluation of its potential benefits relative to existing systems and instruments, accompanied by a comprehensive cost-benefit analysis and assessment of prevailing market structures. Broadly, the advantages of CBDCs can be categorized as improvements in efficiency, support for innovation, and enhancements to the central bank’s functional role.

One often-cited advantage of CBDC is its potential to help monetary authorities overcome the zero lower bound on interest rates (Barrdear and Kumhof 2021). Similarly, commercial banks are likely to contend that deposit-like features embedded in CBDCs could undermine their intermediation role. This concern is compounded by the growing ability of non-financial firms to mimic traditional bank deposit functions, raising the risk of disintermediation (Agur et al. 2022). Furthermore, the merging of monetary and fiscal functions could jeopardize the independence of central banks.

The costs associated with issuing CBDCs differ from those of traditional currency, as they do not include expenses related to handling, printing, storing, or distributing physical cash. Consequently, CBDC issuance could reduce these operational costs and potentially enhance seigniorage, assuming other conditions remain constant. In addition to their conventional role as issuers of money, central banks are increasingly functioning as operators of retail payment systems, engaging directly with market participants and, in some cases, end users. This positioning enables them to spearhead CBDC initiatives with global implications—projects that require leadership, collaboration, and coordination across a broad range of stakeholders, a role that few other institutions are equipped to fulfill (Auer and Böhme 2020).

### Tokenization

Tokenization refers to the process of converting real-world assets, rights, or utilities into digital tokens on a blockchain platform. These tokens can represent a wide array of tangible and intangible assets, enabling more efficient, secure, and transparent mechanisms for managing, trading, and transferring value (Garcia-Teruel and Simón-Moreno 2021).

- Initial coin offerings (ICOs) and token sales: The emergence ICOs marked a pivotal development in the tokenization landscape. ICOs are fundraising mechanisms in which projects issue digital tokens—typically on platforms such as ETH—in exchange for cryptocurrencies like BTC or ETH. These tokens may represent various forms of value, including equity, utility, or access rights. ICOs gained widespread popularity in the mid-2010s as an alternative to traditional venture capital financing, allowing startups and BC projects to raise capital rapidly and with relatively limited regulatory oversight. However, the absence of robust regulation and the prevalence of fraudulent schemes prompted increased scrutiny from regulatory authorities. This led to a decline in ICO activity and the emergence of alternative fundraising models, such as security token offerings and initial exchange offerings. A comparative analysis of these methods was provided by Myalo (2019).
- Tokenization of real-world assets: The tokenization of real estate, art, and commodities represents another key advancement in the DA ecosystem. Doing so enables the fractional ownership of high-value assets, thereby increasing market liquidity, lowering barriers to entry for investors, and facilitating more efficient and accessible marketplaces. Leveraging blockchain technology, tokenized assets can be transferred and traded securely and transparently, creating new opportunities for investment and innovation in financial products. Additionally, tokenization can streamline asset management processes, including settlement, clearing, and auditing. As blockchain infrastructure matures and regulatory frameworks develop, the tokenization of real-world assets is expected to expand and reshape traditional asset markets. An illustrative example of real estate tokenization is available at arcneo.de.

### DeFi

DeFi represents a rapidly expanding segment of the DA ecosystem that utilizes blockchain technology, smart contracts, and decentralized protocols to deliver financial services and applications without the need for traditional financial intermediaries.

1. DeFi platforms and services: DeFi platforms are built atop blockchain networks—most notably ETH—which provide the infrastructure necessary for the development and deployment of smart contracts. These smart contracts are self-executing programs that encode the rules and logic governing financial transactions. They enable the creation of a wide range of DeFi services, including lending, borrowing, trading, and asset management (Schär 2021). Key DeFi services include:
  Decentralized exchanges (DEXs): DEXs enable P2P trading of DAs without the involvement of centralized order books or intermediaries. They utilize automated market makers and liquidity pools to facilitate decentralized and trustless trading (Kokoris-Kogias et al. 2018).
  Lending and borrowing platforms: These platforms allow users to lend and borrow DAs through smart contracts, typically requiring over-collateralization to manage credit risk. Interest rates are algorithmically determined based on supply and demand conditions. A prominent example is aave.com. See also https://quantinar.com/course/174/On-Crypto-backed-Loans.
  Yield farming and liquidity mining: Users can provide liquidity to DeFi protocols by depositing DAs and, in return, earn rewards in the form of native platform tokens. These mechanisms often result in high annual percentage yields (Xu and Feng 2022).
  Derivatives and synthetic assets: DeFi platforms also offer financial instruments such as derivatives and synthetic assets, which mirror the value of underlying traditional assets like stocks or commodities. These instruments enable users to gain exposure to diverse asset classes without holding the actual assets (Zhou et al. 2021).
2. Growth of the DeFi ecosystem: This ecosystem has witnessed exponential growth in recent years, with billions of dollars locked across various DeFi protocols. This expansion is driven by multiple factors, including the proliferation of stablecoins (Saengchote 2021), rising demand for decentralized financial services (Mnohoghitnei et al. 2022), and innovations in DeFi infrastructure (e.g., layer 2 scaling solutions and cross-chain bridges (Darlin et al. 2022)). Nevertheless, the rapid growth of DeFi has also introduced significant risks and challenges, including vulnerabilities in smart contracts, elevated gas fees, and ongoing regulatory uncertainty.

### DA ecosystem risks

**Fig. 3:**
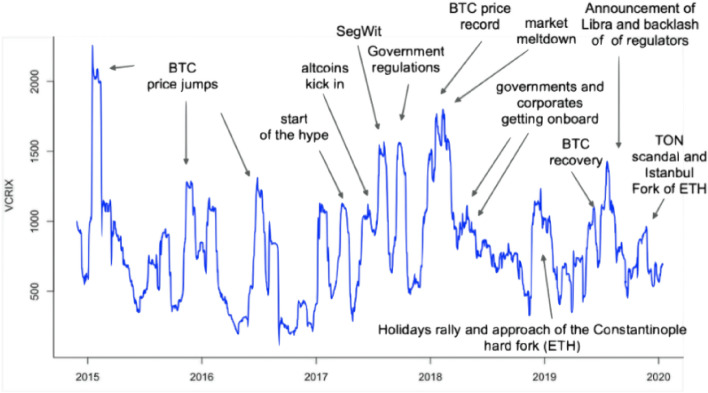
Cryptocurrency Volatility Index (VCRIX) https://quantinar.com/course/61/vcrix

As DAs continue to evolve and gain mainstream adoption, they present various challenges and risks that must be addressed by stakeholders, including regulators, developers, and users. Key challenges and risks include:

1. Technical challenges: DAs face several technical limitations, including:
  Scalability: Many blockchain networks, such as ETH and BTC, encounter scalability constraints, leading to slow transaction throughput and high fees. Layer 2 solutions and alternative consensus mechanisms like proof-of-stake (PoS) are being actively pursued to mitigate these issues (Hafid et al. 2020).
  Interoperability: A lack of interoperability among blockchain platforms and DA ecosystems impedes seamless asset transfers and cross-network transactions. Technologies such as cross-chain bridges and atomic swaps are under development to enhance transactional efficiency and network compatibility (Zhou et al. 2020).
  Security: Ensuring the security of DAs and blockchain platforms is critical, as flaws in smart contracts or consensus algorithms can result in substantial financial losses. Efforts to strengthen the ecosystem include formal verification, the use of secure programming languages, and advancements in cryptographic techniques (Yu et al. 2020).
2. Regulatory and Legal Challenges: The rapid growth and continuous innovation in DAs have outpaced existing regulatory frameworks, resulting in several legal and compliance-related challenges, including:
  Regulatory uncertainty: The absence of clear regulatory classifications and guidelines for DAs introduces ambiguity for developers, investors, and users. This uncertainty can impede innovation and limit the broader development of DA markets (Gulen and Ion 2015).
  {Anti-money laundering (AML)/Know-your-customer (KYC) compliance:} Compliance with AML and KYC regulations presents significant challenges for DA platforms, which must navigate the tension between preserving user privacy and ensuring transparency to combat illicit activities (Norvill et al. 2020).
  Taxation and reporting: DA taxation and reporting obligations are often complex and unclear, posing difficulties for individuals and organizations. There is a pressing need for clearer, more accessible guidelines and streamlined tools to support compliance (Danescu 2020).
3. Market risks: The DA market is inherently volatile, with frequent and often sharp price fluctuations that expose investors and users to significant market risks, including:
  Price volatility: The pronounced price volatility of DAs can result in substantial financial losses for investors and users, particularly in scenarios involving leveraged trading or margin calls. Figure 3 illustrates this volatility by depicting the dynamics of the Cryptocurrency Volatility Index (VCRIX) (https://www.royalton-crix.com/) alongside major events that have influenced the CC market.
4. Liquidity risk: Liquidity levels in DA markets can fluctuate widely, with certain assets exhibiting low trading volumes or substantial bid-ask spreads. Such conditions make it difficult for investors to execute trades at desired prices or exit positions efficiently (Ghabri et al. 2021).
5. Market manipulation: DA markets remain vulnerable to various forms of manipulation, including pump-and-dump schemes and wash trading. These practices can distort price discovery, generate artificial volatility, and mislead investors regarding the true market value of assets (Castonguay and Stein Smith 2020).

## Multifaceted risk structures

### Technological risks

As the contractual mechanisms of DAs grow increasingly complex, investors are exposed to heightened risks stemming from the opaque nature of these digital instruments. In the case of DeFi assets that operate through fully automated transactions, Azar et al. (2022) and Zihan et al. (2023) highlight the challenges investors face in thoroughly evaluating the associated risk characteristics. Currently, the inherent complexity of smart contracts—arising from distributed networks, asymmetric cryptography, and other underlying technologies—makes it difficult to substantiate many claims regarding their actual capabilities and risk profiles (Mik 2017). Establishing and maintaining trust in the technological architecture of DAs, particularly those enhanced by smart contracts, is therefore essential to assuring investors of their reliability. Conversely, flaws or coding errors embedded within opaque contract code can lead to substantial financial harm and significant investor losses. Thus, a lack of transparency in the technical design and operation of DeFi-related DAs represents a critical technological risk factor. A relevant example is R3’s open-source Corda blockchain platform (https://corda.net), which recorded 182 releases within five years of its initial launch in May 2016—averaging roughly one release every 10 days.

In addition to risks arising from contract complexity, a general lack of user familiarity with the technological aspects of blockchain transactions constitutes another major contributor to technology-induced risk for DA investors.

**Fig. 4:**
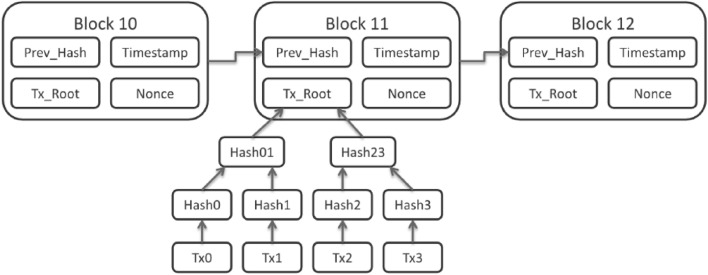
BTC block creation and the “nonce” https://quantinar.com/course/22/crypto

Although blockchain technologies (see Fig. 4) are inherently designed to ensure security and resilience, they are not immune to vulnerabilities. For instance, blockchain networks remain susceptible to Sybil attacks, in which an adversary undermines the reputation system of a P2P network by generating numerous pseudonymous identities to gain disproportionate influence (Douceur 2002). Similarly, blockchain systems may be exposed to routing attacks, where an attacker compromises an internet service provider to manipulate network routes—either partitioning the network or delaying block propagation—which can ultimately result in double-spending incidents (Apostolaki et al. 2017). Accordingly, technological risk factors associated with the DLT underlying certain DAs should be a central consideration in investors’ overall risk assessments.

Furthermore, with the rapid expansion of the DeFi ecosystem and the emergence of DEXs, DAs have become increasingly interconnected with other crypto assets and blockchain networks. The ETH blockchain remains the dominant platform for smart contract deployment (Kaal 2020), and the technological as well as financial stability of its native CC, ETH, therefore carries significant implications for DeFi markets. NFTs, in particular, are exposed to risks inherent in their associated smart contracts. For example, if an NFT represents a digital artwork (see Fig. 2), ownership may be recorded on the blockchain, yet the asset itself could be hosted externally (e.g., on a website). While blockchain records are immutable, external storage systems are not, creating a risk that the DA referenced by a blockchain pointer may eventually become inaccessible or cease to exist.

The risks associated with CCs arise primarily from two dimensions: the underlying platform (typically BC) and their patterns of usage. Roohparvar (2022) identified several relevant risks (Kubicek 2018; Şcheau et al. 2020):

- Crypto-malware and ransomware: Cryptomalware refers to malicious software that exploits unauthorized computational resources to mine CCs. Such malware is commonly introduced through infected websites, advertisements, or compromised trading platforms (Connolly and Wall 2019).
- Use of third-party software: CC investors often rely on third-party tools for a range of functions. However, these tools can introduce vulnerabilities if not properly vetted. Regulatory oversight and the establishment of whitelists for approved applications may help mitigate this risk.
- Unregulated or illegal trading platforms: Many CC trading platforms remain underdeveloped, and their trustworthiness is often uncertain. To date, there is limited regulation ensuring that web-based trading platforms meet established security or reliability standards.
- Phishing attacks: In phishing schemes, attackers impersonate legitimate sources to deceive users and obtain private keys or personal data. Once compromised, attackers can gain full access to victims’ assets (Roohparvar 2022). A recommended countermeasure is the use of secure e-mail gateways that authenticate message sources and filter potentially harmful communications.
- Security of CC accounts: Access to CC accounts is typically secured by passwords that generate private keys in the background. If a password is compromised, recovery is generally impossible. Implementing multi-factor authentication (MFA) provides an additional layer of protection by verifying user identity through multiple credentials (Zhang et al. 2019).
- Unregulated CC exchanges: A defining feature of CCs is their decentralized structure, which, while enhancing autonomy, also limits regulatory oversight. As a result, no central authority currently governs their issuance or circulation. One proposed solution is the establishment of a centralized governing entity—such as a CBDC—responsible for production, management, and oversight of CCs (Chu 2018).
- User perplexity: The technical complexity and specialized terminology of CCs often create confusion among investors, reinforcing perceptions of risk (Saraf and Sabadra 2018). Enhancing accessibility through improved user interfaces, educational resources, and dedicated helpdesks can mitigate these challenges.

A web application firewall (WAF) filters and monitors hypertext transfer protocol traffic between a web application and the Internet to help safeguard web-based systems. WAFs are specifically designed to protect applications against common attack vectors such as cross-site request forgery, cross-site scripting, file inclusion, and SQL injection, among others.

MFA adds an additional layer of protection for DAs by requiring users to verify their identity through multiple authentication factors—typically a password combined with a biometric identifier or a one-time code sent to a registered device.

Digital wallets further enhance DA security by employing encryption and other protective mechanisms to securely store assets and minimize the risk of unauthorized access or theft.

Recent literature has highlighted the growing importance of data privacy and information security in banking and financial services. As financial data and applications become increasingly accessible from remote locations, they face heightened risks of data leakage, privacy violations, and identity theft—threats that are frequently accompanied by potential financial losses.

The Digital Operational Resilience Act (DORA) is an E.U. regulation addressing technological, security, and infrastructural risks within the financial sector (https://www.dora-info.eu). DORA establishes comprehensive requirements for safeguarding the network and information systems of financial institutions, as well as the critical third-party service providers that support them, including cloud platforms and data analytics firms.

### Legal and regulatory risks

Digital-asset risk cannot be separated from *how a token is classified under law*. A “stablecoin” issued in the E.U. may qualify as e-money under the E.U.’s Markets in Crypto-Assets (MiCA) Regulation, a money-market-fund-like product under the U.S. Investment Company Act, or a standard payment token under Singapore’s Payment Services Act. Each classification carries distinct implications for capital requirements, disclosure obligations, and redemption rights. The core regulatory challenge, therefore, lies not in the absence of rules but in the *overlap and collision* of multiple legal frameworks once a token crosses borders or evolves in function. The remainder of this section adopts this perspective to analyze the six classes defined earlier, illustrating how harmonized international definitions can themselves serve as a mechanism for mitigating systemic risk.

The original conception of BTC stands in tension with regulatory authority and government oversight, continuing the cyber-libertarian tradition rooted in John Perry Barlow’s 1996 “Declaration of the Independence of Cyberspace” (https://www.eff.org/ro/cyberspace-independence), which opposed governmental control of online interactions. Nonetheless, while early proponents argued that BTC’s decentralized nature made it impervious to regulation, subsequent developments suggest considerable scope for oversight—and, in certain cases, the potential benefits of regulatory intervention (Böhme et al. 2015).

An illustrative example of market manipulation through simple blockchain transaction ordering highlights the need for more coherent and comprehensive regulatory approaches (Barczentewicz et al. 2023). On public, permissionless blockchains such as ETH, block space is limited, forcing crypto traders to compete for inclusion in transaction blocks. Control over this block space grants validators (i.e., block-producing entities) the capacity to engage in “maximal extractable value” (MEV) strategies, which exploit the discretion to determine the inclusion and sequence of transactions within a block. By reordering transactions to maximize profit (e.g., by allowing certain transactions to “front-run” others), validators can significantly influence market outcomes and generate substantial profits.

Tactics such as “sandwich attacks,,, which involve executing trades ahead of other users’ transactions to capitalize on price movements, have been characterized as toxic, manipulative, and potentially fraudulent—akin to theft. The legality of MEV extraction under U.S. financial law has yet to receive comprehensive scholarly examination, underscoring the fragmented and jurisdiction-specific nature of existing regulatory responses.

#### Regulatory landscape

This section provides an overview of the evolving regulatory landscape. Jurisdictions around the world exhibit a wide spectrum of approaches toward DAs, ranging from highly restrictive environments to more permissive and innovation-friendly regimes. The U.S., for instance, maintains a comprehensive regulatory structure in which DAs are subject to oversight by agencies such as the U.S. Securities and Exchange Commission (SEC), Commodity Futures Trading Commission, and the Financial Crimes Enforcement Network, depending on the asset’s classification and intended use. By contrast, countries like Switzerland and Malta have adopted more supportive frameworks, providing explicit legal guidance that fosters innovation and the development of DA-related business models.

A notable feature of this landscape is the phenomenon of regulatory arbitrage, whereby DA firms may relocate to jurisdictions offering more favorable regulatory conditions in order to sustain or expand their operations.

In examining the regulation of DAs, a key distinction lies in their classification as securities, commodities, or currencies—each category entailing distinct legal and supervisory consequences. DAs identified as securities, including instruments such as ICOs and security tokens, fall under securities law, which typically mandates registration, disclosure, and ongoing reporting obligations. Conversely, DAs categorized as commodities (e.g., BTC and ETH) are regulated under commodity statutes and supervised by corresponding oversight bodies.

The discussion also extends to DAs treated as currencies, particularly stablecoins and CBDCs, assessing their implications for business operations and user engagement. This classification underscores the multidimensional nature of DA regulation, demonstrating the need for nuanced, function-specific oversight mechanisms that reflect the diverse roles DAs play within the global financial ecosystem.

In examining the evolving landscape of DA regulation, this section highlights several influential frameworks and guidelines that are shaping the global DA sector. Among these are the Financial Action Task Force (FATF) guidelines, MiCA, and the oversight activities of the U.S. SEC. Each of these regulatory initiatives plays a pivotal role in establishing compliance norms, safeguarding investors, and maintaining the integrity of DA markets.

The FATF—an intergovernmental organization tasked with combating money laundering and terrorist financing—has issued a series of guidelines that exert significant influence over the operations of virtual asset service providers (VASPs). One of its most consequential measures, the “Travel Rule,” mandates that VASPs collect and transmit identifying information about both senders and recipients of transactions. This requirement enhances transparency within the digital asset ecosystem and reduces opportunities for illicit use, including money laundering and terrorism financing.

Within Europe, the proposed MiCA regulation represents a landmark step toward establishing a unified legal framework for DAs across the E.U. MiCA seeks to harmonize the treatment of crypto-assets, ICOs, and crypto-asset service providers, thereby promoting consumer and investor protection while fostering innovation. By imposing clear standards for disclosure, accountability, and operational conduct, MiCA is expected to play a defining role in shaping how DAs are issued, managed, and traded throughout the EU.

In the U.S., the SEC plays a central role in regulating DAs, particularly those that meet the criteria for classification as securities. The SEC has progressively clarified its position through interpretive guidance, investigative reports (e.g., the “DAO Report”), and enforcement actions against entities that have breached securities laws (e.g., by conducting unregistered ICOs). The Commission’s overarching objective is to safeguard investors from fraudulent activities while ensuring that markets operate in a fair, orderly, and efficient manner. To this end, the SEC closely examines DA exchanges, trading platforms, and broker-dealers to verify compliance with existing securities regulations.

Collectively, the regulatory efforts of the FATF, MiCA, and the SEC reflect a broad, coordinated, and evolving global approach aimed at enhancing clarity, security, and stability within the digital asset ecosystem. By addressing core issues such as anti–money laundering compliance, investor protection, and market integrity, these frameworks are instrumental in shaping the future trajectory of digital asset governance, trading practices, and international regulatory convergence.

Table 3 presents the regulatory approach to digital assets.

**Table 3:** Comparison of jurisdictional regulatory approaches

Jurisdiction	Regulatory approach	Key regulations
United States	Strict Regulatory Environment	SEC, CFTC, FinCEN
Switzerland	Lenient Regulatory Framework	FINMA, Swiss DLT Law
Malta	Lenient Regulatory Framework	MDIA, VFA Act
European Union	Harmonized Framework	MiCA, AMLD5
China	Strict Regulatory Environment	PBOC, MIIT

The regulatory treatment of DAs varies substantially across jurisdictions, resulting in a fragmented and uneven global landscape that introduces significant regulatory risks. One of the primary concerns is the potential misuse of DAs for illicit purposes, including money laundering, terrorism financing, and tax evasion. In response, many countries have implemented KYC and AML requirements for DA exchanges and other VASPs to enhance transparency and accountability.

Beyond financial crime prevention, regulators face additional challenges related to investor protection, market integrity, and financial stability. Approaches differ widely: while some jurisdictions have adopted a more laissez-faire stance to encourage innovation, others have introduced licensing and registration regimes for businesses operating in the DA sector. This diversity of approaches underscores the need for greater international coordination to balance innovation with effective oversight.

Although NFTs are not yet subject to specific regulation, legal obligations may still be imposed by national or international authorities. Such obligations could include KYC procedures, verification requirements concerning the authenticity and nature of the assets being sold, as well as record-keeping and broader AML compliance measures. These may extend to sanctions enforcement or forthcoming frameworks such as MiCA.

The precise legal status of NFTs remains unresolved, and uncertainty persists regarding whether certain NFTs could qualify as securities under existing Financial Conduct Authority guidance. At present, a regulatory vacuum exists, leaving NFTs without comprehensive oversight or a unified supervisory framework.

The need for greater regulatory clarity surrounding NFTs has been acknowledged by the intergovernmental FATF. In its most recent guidance, the FATF provides direction to regulators on when and how NFTs should be identified and treated as virtual assets.

According to this guidance, NFTs are not automatically classified as virtual assets based solely on their general characteristics. Instead, regulators are advised to assess each case individually. The FATF stipulates that NFTs should be regulated as virtual assets only when their use aligns with the functional definition of a virtual asset.

As the FATF noted, “some NFTs that, on their face, may not appear to constitute virtual assets could nevertheless fall within the definition if, in practice, they are used for payment or investment purposes.” This principle underscores the need for a functional, use-based approach to classification rather than one based solely on form.

The FATF advocates for a functional approach to NFT regulation, emphasizing that classification should be based on an asset’s actual use rather than the underlying technology. The key challenge for stakeholders involved in NFT markets is therefore to determine which NFTs satisfy the FATF’s functional definition of a virtual asset. Once an NFT meets this definition, the corresponding compliance, monitoring, and reporting obligations must be fulfilled.

Meeting these obligations necessitates the adoption of a risk-based approach, aimed at preventing illicit or high-risk transactions and ensuring that suspicious activities are promptly identified and reported. Regulators are likely to expect proactive risk management practices from entities engaged in NFT-related activities, reflecting a broader shift toward accountability and due diligence in the DA sector.

#### DA adoption

The adoption of DAs has expanded substantially, with a diverse range of stakeholders—including institutional investors, retail participants, and industry actors—increasingly integrating DAs into their investment strategies and operational frameworks.

1. Institutional Adoption:
  Custody solutions: The development and deployment of secure, compliant custody mechanisms (e.g., multi-signature wallets and hardware security modules) have enabled traditional financial institutions to safely store and manage DAs on behalf of their clients.
  Trading and execution: DA trading has been incorporated into existing trading infrastructures, alongside the emergence of dedicated institutional-grade platforms. These systems utilize algorithmic trading and smart order routing to enhance execution quality and optimize risk exposure (Al-Shaibani et al. 2020; Haberly et al. 2019).
  Risk management: Institutions are adopting sophisticated risk management frameworks, including value-at-risk (VaR) models, stress testing, and portfolio optimization tools, to identify, measure, and mitigate the unique risks inherent in DA markets (Pele et al. 2021).
  Asset tokenization: The tokenization of conventional financial instruments (e.g., equities, bonds, and real estate) facilitates fractional ownership, increases market liquidity, and broadens access for a wider base of investors (Cong et al. 2020).
2. Retail Adoption:
  Exchanges and brokerages: The emergence of intuitive and secure trading platforms has enabled retail investors to buy, sell, and exchange DAs with ease. These platforms often incorporate security features such as two-factor authentication, insurance coverage, and fiat on-ramps to facilitate accessibility and trust (Dai et al. 2020).
  Wallets and storage: A variety of wallet solutions—ranging from hardware and desktop software to mobile applications—have been developed to enhance user experience, security, and compatibility across multiple DAs (He et al. 2020).
  Payment solutions: DAs are increasingly being integrated into payment infrastructures, allowing individuals to conduct everyday transactions, remittances, and cross-border payments. Technologies such as the Lightning Network and stablecoins play a key role in improving transaction efficiency and scalability (Varma and Maguluri 2019).
  Financial services: The growth of DA-based financial products and services (e.g., lending platforms, staking mechanisms, and yield-farming opportunities) enables retail users to earn returns on their digital holdings and participate more actively in DeFi ecosystems (Xu and Feng 2022).
3. Use Cases and Industry Applications:
  Supply chain management: Blockchain technology is increasingly employed to enhance supply chain transparency, traceability, and operational efficiency by establishing immutable records of product provenance and transaction histories.
  Identity management: DAs and BC frameworks support the development of secure, decentralized identity management systems, allowing individuals to maintain greater control over their personal data while streamlining access to digital services.
  DeFi: The rapid expansion of DeFi platforms and protocols has enabled a range of services—including lending, borrowing, insurance, and derivatives trading—delivered through smart contracts and powered by DAs.
  Digital collectibles and NFTs: The rise of digital collectibles and NFT facilitates the tokenization of unique digital and tangible assets, fostering new applications across art, gaming, entertainment, and other creative industries.

#### MiCA regulation

MiCA represents the E.U.’s comprehensive legal framework governing the issuance, classification, and supervision of crypto assets. It specifically regulates issuers of stablecoins—distinguishing between asset-referenced tokens and electronic money tokens—while also encompassing all other categories of crypto assets. Adopted by the European Parliament on April 20, 2023, MiCA stands as the first legislation of its kind globally, setting a precedent for other jurisdictions.

The regulation was initially driven by the emergence of Facebook’s Libra project, which underscored the need for regulatory clarity around stablecoins. MiCA introduces a clear taxonomy of DAs, providing standardized criteria for differentiating between asset classes. It also establishes a supervisory framework for crypto-asset service providers, covering activities such as trading, custody, and investment advisory services related to crypto assets.

Published in July 2023, MiCA is being implemented in two phases: a 12-month transition period for provisions concerning stablecoins, followed by an 18-month phase-in for the remaining aspects of the regulation. As such, MiCA marks a foundational step toward harmonized crypto-asset regulation within the E.U. and beyond.

#### Navigating the complexities of compliance

The novel risks introduced by financial technologies (FinTech) necessitate equally innovative regulatory responses, often described as “Smart Regulation,” supported by regulatory and supervisory technologies collectively referred to as “RegTech.” The term RegTech, derived from “regulatory technology,” encompasses the application of information technology to enhance compliance, monitoring, reporting, and oversight processes.

Practical examples of RegTech include algorithmic systems that analyze trading patterns in listed securities to detect potential insider trading, as well as electronic KYC solutions that streamline client onboarding while reinforcing transparency and market integrity. These tools demonstrate how technology can both improve regulatory efficiency and strengthen safeguards across digital financial ecosystems.

In the E.U., DAs—including CCs—are subject to AML, counter-terrorism financing, taxation, and reporting requirements comparable to those applied to traditional financial instruments. For VASPs operating within the E.U., the “Fifth Anti–Money Laundering Directive,” which came into effect in January 2020, introduced specific compliance obligations designed to enhance transparency and reduce financial crime risks.

#### Regulatory challenges and opportunities

This section outlines the principal challenges and opportunities associated with regulating DAs from a technical and policy perspective.

- Balancing innovation and risk: Protecting consumers and investors from fraud, market manipulation, and cyber threats requires a regulatory architecture that mitigates risks while preserving the capacity for technological innovation (Poshan et al. 2022).
- Ensuring market integrity: Developing mechanisms to uphold fair, transparent, and efficient markets necessitates robust surveillance tools capable of detecting manipulative behavior and sustaining investor confidence and market stability (Demmou and Sagot 2021).
- Promoting financial stability: Identifying and managing potential systemic risks posed by DAs to monetary policy and financial stability remains a central regulatory objective (de Koker et al. 2019; Hussain et al. 2020; Usman Bashir and Hussain 2020).
- Global regulatory coordination: The effective governance of DAs depends on harmonizing regulatory standards and fostering cross-border collaboration to address the inherently global nature of digital asset markets (Allen et al. 2020b).
- Regulatory sandboxes and innovation hubs: Establishing supervised environments where emerging technologies and business models can be tested safely supports both regulatory adaptation and sustainable sectoral growth (Stavrova 2021).

These studies emphasized the necessity of a balanced, coordinated, and adaptable regulatory approach to DAs—one that ensures consumer and investor protection, promotes market integrity, and fosters the sustainable development of the broader digital asset ecosystem.

However, more nuanced attention must be given to the regulation of NFT, which raise complex legal considerations encompassing intellectual property rights, contractual obligations, and consumer protection. The treatment of NFTs under national laws, such as in Germany, illustrates the fragmented and jurisdiction-specific nature of current regulatory approaches. Under German law, tokens are classified as crypto assets only if they are recognized as a means of exchange or payment, or if they serve investment-related purposes.

BaFin, Germany’s Federal Financial Supervisory Authority, assesses NFTs according to the issuer’s stated expectations. If an issuer promotes the potential for value appreciation as a primary selling feature, BaFin may classify the NFT as a regulated crypto asset. In other cases, NFTs may be categorized as asset investments under the German Asset Investment Act, depending on their structure and purpose.

Given the diversity of NFT applications, regulators must pursue sector-specific approaches rather than adopting a uniform, one-size-fits-all framework. A generalized approach risks overlooking important distinctions, such as taxable property consideration as NFTs often fall outside existing tax frameworks. For instance, while the United States treats cryptocurrencies as taxable property, NFTs may present exceptional cases.

Security concerns also merit particular attention. NFT systems depend on technical components that are vulnerable to threats such as “spoofing,” in which attackers steal authentication credentials, and “tampering,” where off-chain data linked to NFTs can be altered or deleted. Furthermore, as NFT ecosystems evolve, the need for interoperability across multiple blockchains introduces additional challenges. Cross-chain communication mechanisms, while necessary for integration, may inadvertently undermine decentralization or expose systems to new attack vectors.

#### Competing scholarly perspectives

Recent scholarship reveals the emergence of distinct normative perspectives concerning the risks associated with crypto assets and the appropriate scope of regulatory intervention. The principal viewpoints are summarized in Table 4.

One school of thought maintains that crypto markets exhibit self-correcting mechanisms, arguing that minimal regulation is preferable to encourage innovation, market experimentation, and technological advancement. In contrast, another camp views crypto assets as potential sources of systemic risk comparable to those observed in shadow banking, emphasizing the need for robust oversight to safeguard financial stability and protect investors.

A third, more moderate position advocates for regulatory frameworks that enhance transparency and facilitate real-time market discipline while avoiding excessively prescriptive or restrictive measures. This approach seeks to balance innovation with accountability, ensuring that regulatory objectives are met without unduly constraining the growth of digital financial markets.

**Table 4:** Competing scholarly perspectives on crypto-asset regulation

Perspective	Main arguments	Representative sources
Techno-libertarian	Crypto markets are self-correcting; regulation should be minimal to support innovation	Cato Institute (2025)
Prudential-centric	Crypto creates systemic risks similar to shadow banking; strong regulation needed to protect stability	Gorton and Metrick (2010)
Market-disciplinarian	Regulation should promote transparency and real-time market discipline without heavy-handed intervention	Koenraadt and Leung (2024)

#### NFT legal doctrine

NFTs present intricate classification challenges within financial and securities law. In the United States, courts commonly apply the Howey Test to determine whether an NFT constitutes an investment contract. This assessment examines whether there is an investment of money, in a common enterprise, with a reasonable expectation of profits, derived from the efforts of others. Recent regulatory interpretations suggest that NFTs offering fractionalized ownership interests, profit-sharing arrangements, or active promotional campaigns may meet the criteria for classification as securities.

MiCA formally excludes NFTs from E.U.’s scope. However, interpretive complexities emerge when NFTs are marketed as fungible series or combined with financial features, such as collective investment or yield mechanisms (Inozemtsev 2021a). National authorities have adopted varying approaches: Germany evaluates NFTs under existing financial instrument definitions where rights or claims are attached, while France applies its consumer protection and AML regimes to address emerging risks.

Across Asian jurisdictions, including Singapore and Japan, regulatory analysis tends to be case-specific, emphasizing the functional attributes of NFTs—particularly when financial returns or pooling structures are present. Persistent areas of doctrinal ambiguity include the classification of dynamic NFTs, the regulatory perimeter for NFT marketplaces, and their treatment within DeFi ecosystems. A more systematic legal engagement with these interpretive questions would strengthen the analytical foundation for assessing NFT-related risks (Inozemtsev 2021b).

### Market risks

#### Volatility and downside risk

DAs represent a distinct asset class that offers investors both opportunities and challenges, with associated risks determined by characteristics such as volatility, type, and underlying structure. Among these, CCs have emerged as the most liquid and actively traded category, maintaining this status for nearly a decade. Despite their liquidity, CCs remain subject to pronounced price fluctuations and speculative dynamics. Investors must therefore carefully assess both the potential returns and inherent risks before engaging in this market (Hussain and Bashir 2020). In this section, we focus on CCs and analyze their evolution with particular attention to tail-event behavior. The tail properties of the profit-and-loss distribution play a crucial role in shaping the risk structure of CC-based portfolios and are effectively captured through spectral risk measures (SRMs).

Exponential and power SRMs, originally developed by Dowd et al. (2008), are among the most widely applied approaches in this context. Prior studies have demonstrated that the exponential utility function may serve as a reasonable choice under specific conditions (Bühlmann 1980). Nonetheless, the appropriate selection of the utility function and risk-aversion parameter must reflect the counterparty’s risk preferences and the portfolio’s strategic context. In Lu et al. (2025), SRM-based risk structures were evaluated across various portfolio configurations.

Empirical analysis reveals that during periods of heightened market turbulence, such as the COVID-19 pandemic, portfolios characterized by higher volatility tend to exhibit more extreme returns. When assessing minimum exponential SRM portfolios across parameter values ranging from 1 to 25, it is observed that portfolios with a parameter value of $k=1$, indicating minimal risk aversion, display the greatest volatility in cumulative wealth. Conversely, the portfolio associated with $k=20$, representing a more risk-averse stance, delivers superior performance.

**Fig. 5:**
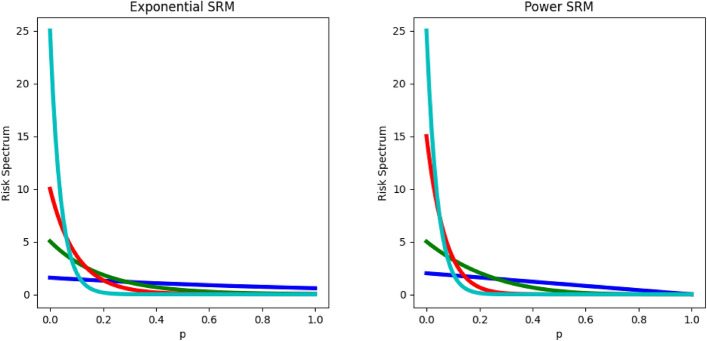
Risk spectra for different SRMs—Left: $k=$; Right: $\gamma =$https://github.com/QuantLet/SRMforDA/tree/main/SRMforDA_RiskSpectrum

A similar examination using the power SRM approach considers two distinct market scenarios. The results suggest that as SRM values decline across these cases, portfolio turnover also decreases. This finding indicates that strategies with lower turnover are less sensitive to transaction costs and thus more likely to sustain improved long-term performance (Fig. 5).

#### Risk contagion

A key issue of both practical and academic relevance is understanding the dependence structure among digital securities, as it directly informs strategies for market risk diversification. Investors in CCs have repeatedly experienced pronounced volatility and extended drawdown periods, most notably during the 2021 market crash.

Kwapień et al. (2021) investigate the cross-correlation structure of returns across a broad panel of 80 CCs traded on the Binance exchange. Using *q*-dependent detrended cross-correlation coefficients and spectral analysis of the correlation matrix, the study finds that the CC market has progressively become more compact and interdependent. Correlations among virtual coin returns intensify during episodes of financial stress (e.g., years immediately following the onset of the COVID-19 pandemic) and tend to weaken during relatively stable periods.

Building on this perspective, Agyei et al. (2022) applied wavelet methods to examine both synchronous and asynchronous linkages between commonalities in CC returns and the VCRIX. As a forward-looking indicator, VCRIX captures investor sentiment and perceived uncertainty regarding future CC market movements. Drawing on a comprehensive daily dataset spanning August 2017 to August 2021—including major global disruptions such as the U.S.–China trade conflict and the COVID-19 crisis (Wang et al. 2022)—the authors documented a strong relationship between CC returns and VCRIX across multiple investment horizons. Their results also revealed a significant correlation between BTC and other major cryptocurrencies, implying limited diversification potential within portfolios composed solely of CC assets. Nonetheless, a portion of volatility in CC markets appears to originate from idiosyncratic, non-systematic factors unrelated to VCRIX fluctuations. This suggests that while individual CC investments remain highly risky, diversified portfolios of CCs may provide improved risk control under specific market conditions.

Further, James and Menzies (2022) examined the interplay between collective market dynamics, asset size, returns, and volatility within the CC ecosystem. Their findings indicated that interdependencies among CC prices intensify during bearish market regimes, while correlations decline during recovery phases. The study also identifies a positive association between volatility and market capitalization, suggesting that CCs of similar size exhibit comparable volatility patterns, thereby reinforcing the market’s tendency toward regime-dependent integration.

At this point, it is important to recognize that CCs currently constitute only a minor component of most institutional portfolios. For the majority of fund managers, CCs serve as supplementary instruments within broader investment strategies that remain dominated by traditional financial assets such as equities, bonds, foreign exchange, and commodities. The financial literature increasingly suggests that it has become progressively more difficult for global investors to achieve meaningful diversification among conventional asset classes. This challenge arises from the growing correlations among returns, particularly during periods of market stress. Nonetheless, when virtual coins exhibit low or negative correlations with traditional assets, they may enhance the overall risk–return profile of a diversified portfolio.

This issue has been explored in several empirical studies. BenSaïda (2023) examine the relationship between BTC exchange rates and government-issued currencies across a large cross-section of developed and emerging economies. Using a copula-based approach, the authors identify a time-varying dependence structure in which cross-market linkages intensify during episodes of economic distress or heightened volatility in virtual coin markets. Their findings highlight particularly strong coupling during recent crises, such as the 2021 BTC crash and the 2022 Russia–Ukraine conflict, signaling a growing alignment between virtual and sovereign currencies.

Similarly, Charfeddine et al. (2020) employed copula techniques to assess tail dependencies between BTC, ETH, and traditional financial assets in order to evaluate diversification potential. The results reveal a relatively weak and time-varying dependence structure between these CCs and conventional securities, suggesting that under certain conditions, cryptocurrencies can provide limited but meaningful opportunities for portfolio diversification.

Pele et al. (2021) examine the relationship between virtual coins and traditional investment asset classes, including stocks, bonds, real estate, and commodities. Using dimensionality reduction and classification techniques, they identify three latent factors that jointly explain daily log returns across the analyzed asset panel: a tail factor, a memory factor, and a moment factor. CCs display particularly strong exposure to the tail factor, distinguishing them from conventional assets. They also exhibit a notable degree of internal coherence, meaning that they tend to cluster together as an asset class and periodically diverge from traditional financial assets.

Expanding on this line of research, Ahn (2022) investigated extreme co-movements between CC and equity returns. Using a model-free approach, the authors find strong left-tail dependence between returns on major CCs and the S&P 500 index, suggesting that extreme negative shocks often occur simultaneously across both markets.

Similarly, Jiang et al. (2021) assessed the diversification potential of six major CCs relative to six large-cap stock indices. Applying a quantile coherency framework, they show that CC returns are generally positively correlated with stock market movements, with stronger alignment observed over medium- and long-term horizons—particularly during periods of market downturn. This evidence implies that CCs may offer limited diversification benefits when equity markets experience stress.

The two aforementioned studies highlight the limited opportunities for risk diversification provided by virtual coins, particularly during periods of equity market decline. Maghyereh and Abdoh (2020) examine BTC’s linkages with a broader range of asset classes, including stocks, bonds, currencies, and commodities. Their analysis identifies significant long-term dependencies between BTC and the S&P 500, while its relationship with the USD–EUR exchange rate appears comparatively weak. The results further suggest that fluctuations in traditional asset prices Granger-cause BTC returns in lower quantiles, although the reverse causal relationship is generally not observed.

Evidence also indicates that volatility contagion among CCs is stronger within the CC market itself than between CCs and broader risk indices such as the VIX, Crude Oil Volatility Index, Economic Policy Uncertainty Index, or Geopolitical Risk Index (Al-Yahyaee et al. 2019). Furthermore, Dong et al. (2022) find that many of the anomalies observed in CC markets become more pronounced during periods of low liquidity, underscoring the sensitivity of CC market behavior to liquidity conditions.

There is substantial empirical evidence in the literature concerning the market risk profile of DAs and their comparative performance relative to traditional asset classes. Existing research consistently indicates a high degree of integration among various virtual coins. Risk contagion within the CC market is particularly pronounced, implying that opportunities for diversifying away coin-specific risks within crypto-heavy portfolios remain limited.

Intercorrelations among CCs tend to intensify during bearish market regimes, suggesting that under such stressed conditions, investors should rely on derivatives and other hedging instruments available within the virtual-asset ecosystem to manage overall risk exposure. However, the limited liquidity of these instruments, combined with market frictions such as transaction costs and margin requirements, may constrain the practical effectiveness of such hedging strategies. In this context, regulatory authorities could play a crucial role by implementing measures aimed at improving market depth, transparency, and efficiency to enhance the resilience of risk management practices in DA markets.

The literature provides mixed evidence regarding the degree of coupling between returns on virtual coins and other classes of financial securities. The correlation structure appears asymmetric; in bullish market conditions, DA prices are largely influenced by asset-specific developments and news, whereas during periods of market turbulence, their returns tend to be overshadowed by the downward movements of major market indices. Given this empirically observed feature (i.e., left-tail dependence), a global diversification strategy (allocating capital across asset classes and countries) may offer limited effectiveness in hedging against shocks originating in the DA market.

It also remains uncertain whether DAs can serve as a valuable complement to institutional portfolios by improving the risk–return profile of traditional asset classes, particularly during times of underperformance. Moreover, global stablecoins present elevated risks to financial stability due to their cross-jurisdictional nature and potential for large-scale adoption. These risks may be further amplified if GSCs evolve into major stores of value, potentially inducing volatility within key payment and settlement systems (Financial Stability Board and International Monetary Fund 2023).

The Financial Risk Meter (FRM; https://theIDA.net/) has emerged as an important analytical instrument for assessing and managing the distinctive and volatile risk environment of cryptocurrencies (Ren et al. 2025). Utilizing advanced statistical techniques—most notably quantile lasso regression—the FRM systematically monitors systemic risk and contagion effects within crypto asset markets (https://ida.ase.ro/financial-risk-meter/frm-crypto/). This methodology underscores the high degree of interconnection among DAs and provides both investors and policymakers with a detailed understanding of evolving risk dynamics. Such insights are crucial for designing effective risk mitigation strategies and safeguarding financial stability in this fast-changing domain.

**Fig. 6:**
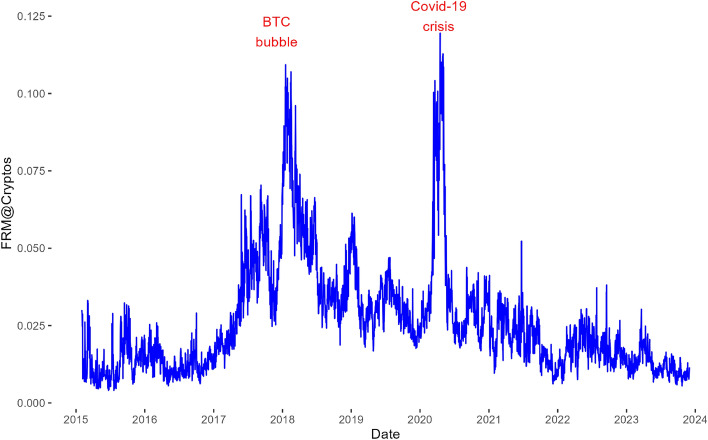
FRM cryptos https://quantinar.com/course/47/frmcrypto

As illustrated in Fig. 6, FRM crypto effectively captures the pronounced volatility of the CC market, including the sharp downturn in 2018 that followed the explosive growth of 2017. It also reflects the subsequent fluctuations in BTC prices toward the end of 2018, signaling elevated systemic tail-event risk during these turbulent periods.

#### Volatility and extreme events

A key criticism of CCs as a means of payment lies in their exceptionally high volatility relative to fiat currencies. For instance, while the annualized volatility of the EUR/USD exchange rate typically ranges between 10 and 15%, BTC’s volatility can rise to several hundred percent per year—an order of magnitude inconsistent with the stability expected of a reliable store of value. Nevertheless, several studies have documented a gradual long-term decline in crypto volatility, particularly among assets with greater market adoption and capitalization.

A related concern involves the heightened likelihood of extreme events—large market shocks that occur far more frequently in DAs than in traditional financial instruments (see Fig. 3). Risk metrics such as VaR and expected shortfall tend to rise sharply in response to the fat-tailed nature of DA return distributions, especially during volatile periods. Furthermore, episodes of heightened turbulence are often accompanied by simultaneous increases in volatility and cross-asset correlations among cryptocurrencies, thereby eroding the diversification benefits within portfolios. Effective market risk management in such environments thus requires adaptive, dynamic measures that capture both linear and nonlinear dependencies across assets.

For NFTs, it is inherently difficult to assess market risk a priori, as these assets are highly heterogeneous. A prerequisite for analysis is the construction of an index that adequately represents the broader market or a specific segment within it (e.g., DAI; Lin et al. 2022). After such an index is established, standard quantitative methods can be employed to evaluate volatility, analyze correlations with other markets, and derive relevant risk measures (see https://quantinar.com/course/148/dai-digital-art-index).

### Liquidity risk and market stability

Market liquidity is a fundamental component of well-functioning financial systems and a primary focus for investors, regulators, and supervisory authorities alike. As noted by Borio (2000), liquidity—much like systemic risk—is often easier to recognize than to define. Illiquidity typically arises from underlying market imperfections, including participation and transaction costs, asymmetric information, limited competition, funding constraints, and search frictions (Bashir et al. 2021).

Vayanos and Wang (2013) provided both theoretical and empirical insights into the determinants of market liquidity. Their study addresses three central questions: how to assess illiquidity, how it relates to structural market imperfections and asset characteristics, and how it influences expected asset returns. Assets with lower liquidity tend to exhibit higher liquidity risk premia, wider bid–ask spreads, and elevated transaction costs. Post-crisis reforms have improved the pricing of liquidity risk premia, thereby mitigating the rapid proliferation of instruments with opaque risk profiles. Nonetheless, persistent shortages of liquidity in certain asset classes can outweigh the benefits of financial innovation for market participants and end users.

Market participants, central banks, and regulatory authorities focus on market liquidity for two main reasons: one linked to the long-term development of the financial system, and the other related to short-term market dynamics. Since the 1970s, the rapid expansion and sophistication of financial markets have reshaped global financial systems. As a result, market liquidity has become increasingly significant for maintaining monetary and financial stability, particularly as central banks have shifted toward market-oriented operational frameworks and the use of asset prices as policy indicators.

Asset prices serve as valuable signals for policymakers, reflecting how market participants perceive and price risk. The reliability of this information enhances market discipline—an objective that remains a central concern of modern financial governance. Moreover, as financial institutions have become more dependent on market mechanisms for risk management, ensuring robust and resilient liquidity during periods of market stress has become critical. Importantly, the depth and stability of liquidity are now closely tied to institutional risk management practices and the overall structure of market participation.

Liquidity-focused investors often seek straightforward metrics that can be derived from daily trading data. This has led to a range of volatility and liquidity studies, such as those by Amihud (2002), Kyle and Obizhaeva (2016), Corwin and Schultz (2012), and Abdi and Ranaldo (2017). According to Brauneis et al. (2021), the first two proxies tend to outperform others in estimating overall liquidity levels, while the measure proposed by Corwin and Schultz (2012) provides superior performance in capturing time-series variations. The latter approach requires four key price points (i.e., high, low, open, and close) whereas the former relies solely on closing prices and trading volumes.

Even when volume data are partially missing, researchers frequently depend on the Amihud illiquidity measure as a reliable proxy (Long et al. 2022). However, Brauneis et al. (2021) highlighted a major concern regarding the reliability and consistency of data provided by certain CC exchanges.

Although the overall size of the crypto asset market remains relatively modest compared to the global financial system, and banks’ direct exposures to crypto assets are limited, the sector nonetheless poses potential financial stability challenges and significant risks for banking institutions (see https://www.bis.org/bcbs/publ/d490.pdf).

Regulatory authorities emphasize the importance of sound liquidity risk management and recommend the implementation of the following key controls:

- Identify the main factors influencing potential deposit behavior to determine which deposits are most vulnerable to volatility.
- Evaluate concentration risks and interconnections among crypto-related deposits to assess their implications for overall liquidity exposure.
- Integrate liquidity risks and funding volatility into contingency funding plans, including liquidity stress testing and robust asset–liability management practices.
- Conduct thorough due diligence and maintain ongoing monitoring of crypto-asset-related entities on a regular basis to ensure continued risk awareness.
- Ensure that banking institutions fully comply with applicable laws and regulations, including brokered deposit provisions and call report filing requirements.

Several characteristics of the DA ecosystem contribute to vulnerabilities that may threaten overall financial stability. For instance, automation can heighten operational risks, as the reduced response time for human intervention increases the likelihood of rapid and self-reinforcing asset sell-offs. Furthermore, the high degree of interconnection among blockchains, cryptocurrencies, stablecoins, DeFi protocols, and centralized exchanges allows shocks in one area to spill over swiftly into others, amplifying systemic risk.

### Socioeconomic risks

#### Unequal access to digital financial services

The disparity between individuals who have access to information and communication technologies and digital resources and those who do not is commonly referred to as the “digital divide.” Socioeconomic determinants—including income, education, gender, geographic location, and technological infrastructure—continue to perpetuate this divide. It remains particularly pronounced between urban and rural areas, across high- and low-income countries, and among populations with differing educational attainment (Du Preez and Le Grange 2020).

**Fig. 7:**
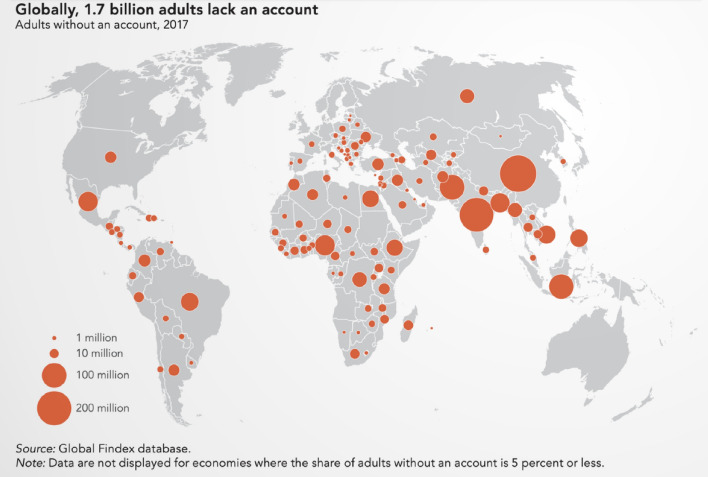
Visual representation of the global digital divide

Not only is slower internet connectivity a persistent concern, but as of 2022, approximately 2.7 billion people—around one-third of the global population—remain unconnected to the internet, according to the International Telecommunication Union’s “Facts and Figures 2022” report (www.itu.int). Moreover, the “Deloitte Global Mobile Consumer Trends” report (2020) indicates that smartphone penetration has reached 80% in developed economies and 82% in developing ones. Despite these advances, this still leaves about 20% of individuals unable to access services that are exclusively available on smart devices, thereby reinforcing the digital divide. Figure 7 provides a visual representation of the global disparity in digital connectivity.

Friedline et al. (2019) shed light on the relationship between digital inequality, the digital divide, and the adoption of FinTech, finding that early adopters are generally younger individuals with higher digital literacy, residing in urban areas, and possessing higher incomes. In a related study, Friedline et al. (2021) observed that communities with larger proportions of Black, Latinx, and American Indian/Alaska Native populations tend to exhibit lower rates of FinTech usage. This dynamic risks excluding indigenous and minority populations from financial inclusion, particularly those who may be illiterate or semi-literate (Senyo and Osabutey 2020).

#### Regime shifts

Examining the evolution of market and regulatory conditions during expansionary phases provides valuable insights into potential future risks. For instance, during the European sovereign debt crisis (2010–2012), which followed the global banking crisis of 2007–2009, policymakers reached a consensus to maintain low short-term interest rates and extend jointly funded public loans to countries that had lost access to primary markets. In 2015, the European Central Bank launched a quantitative easing program aimed at strengthening the transmission of monetary policy through capital markets. While this program helped narrow credit spreads among European nations and pushed long-term interest rates into negative territory, it failed for an extended period to generate sustained inflationary pressures.

The regime shift in interest rates and inflation only materialized in 2022. Many investors had long regarded the preceding low-interest-rate environment as a form of financial repression. During this period, unregulated investors increasingly turned to CCs, attracted by their unbounded structure and potential for exponential returns despite their limited intrinsic value. One explanation for the prolonged absence of CC regulation lies in their classification as currencies rather than securities. Currencies, typically issued by foreign central banks, are not subject to domestic securities laws, whereas securities are regulated across multiple dimensions—covering product design, market conduct, distribution, and the financial service provider’s operations.

A second explanation is the broader fascination with CCs’ technology-driven architecture and their promise of decentralized operation, independent from large financial institutions or state oversight. This vision particularly appeals to libertarian perspectives that reject the notion of state-backed fiat money, even when such backing is supported by taxation authority.

With the regime shift to rising interest rates in 2022, CC markets exhibited stronger correlations with equity markets and experienced substantial declines. This downturn coincided with a series of high-profile fraud revelations, such as FTX and Terra Luna (https://time.com/6243618/crypto-lessons-for-2023/), and intensified enforcement actions by the SEC within the U.S. crypto sector. A BIS study https://www.bis.org/publ/bisbull69.htm shows most retail investors lost money with their crypto investments. In the US, African American lost more on a relative basis than white people https://www.economist.com/graphic-detail/2022/05/20/why-the-crypto-crash-hit-black-americans-hard, https://www.ft.com/content/47d338e2-3d3c-40ce-8a09-abfa25c16a7f.

In an effort to regain investor confidence, major crypto exchanges have sought to enhance transparency by publishing “proof of reserves” https://www.coalexander.com/post/how-should-binance-prove-it-is-solvent. However, transparency alone cannot substitute for comprehensive stress testing. The collapse of Silicon Valley Bank in March 2023, stemming from flawed asset–liability management practices, underscores the limits of disclosure without robust resilience testing.

Beyond concerns about the solvency of trading platforms and the absence of effective market oversight, a more fundamental issue persists: the lack of intrinsic value in many so-called DAs. Assets not linked to a real-world reference or underpinned by a functional utility token remain speculative by nature. From a valuation standpoint, standard asset pricing models would yield a fair value of zero, as the sum of expected discounted cash flows is effectively null. For many DAs, scarcity is presented as the primary justification for value. While scarcity can be enforced technologically at the level of an individual asset, it fails to hold across the broader digital landscape, since new DAs can be created indefinitely. This undermines any cross-sectional notion of scarcity and challenges the sustainability of value claims within this market.

### Environmental risks

The energy required for computation and transaction validation generates a considerable carbon footprint for many DAs. Much of this energy originates from coal-powered plants, as mining activities are typically concentrated in regions offering the lowest electricity costs worldwide.

Two primary consensus mechanisms govern CCs: PoW and PoS. The former entails significantly higher energy consumption than the latter, meaning that different coins exhibit varying degrees of environmental impact (Jones et al. 2022). A notable example of mitigation efforts is ETH’s transition from PoW to PoS, commonly referred to as *The Merge*, which substantially reduced its energy intensity.

Further initiatives are also emerging. Wang et al. (2022) introduced the Cryptocurrency Environmental Attention Index (CEAI), derived from media headlines highlighting environmental risks. This index tracks investors’ awareness of ecological concerns and has shown a marked upward trend since 2021. In parallel, the Cambridge Centre for Alternative Finance maintains the Cambridge Bitcoin Electricity Consumption Index, providing near-real-time estimates of BTC’s energy use (University of Cambridge 2023). While actual electricity consumption cannot be measured directly, the index offers a theoretical range—from minimum to maximum possible expenditure—serving as a useful benchmark for assessing the environmental burden of mining activities.

These initiatives would benefit from greater coordination. A comprehensive monitoring framework for energy consumption within the E.U. should be established—one that not only tracks BTC but also encompasses other major cryptocurrencies. Equally important are educational efforts aimed at raising investor awareness regarding the environmental consequences of PoW-based currencies.

Environmental exposure remains an underexplored factor in academic studies on portfolio construction and hedging strategies. Notable exceptions include Ren and Lucey (2022), who compared PoW and non-PoW crypto-assets as potential safe-haven instruments against clean energy assets represented by green indices. Their findings suggest that clean energy indices serve as safe havens primarily for “dirty” coins. Similarly, Umar et al. (2022) investigated the interconnectedness between clean and dirty assets and the CEAI, concluding that CEAI exerts a stronger influence on equities than on bonds. Bȩdowska-Sójka and Kliber (2024) demonstrated that indices composed of clean energy sources provide better hedging performance against oil (a “dirty” energy source) than either clean or dirty CCs, largely due to the latter’s high volatility. Furthermore, Woitschig et al. (2023) analyze ETH’s energy consumption, while Lin et al. (2020) assessed the environmental impact of its transition to PoS through “The Merge.”

## Mitigating DA risks

To mitigate the risk of cyberattacks, organizations and governments should invest in comprehensive cybersecurity frameworks that include firewalls, antivirus software, intrusion detection and prevention systems, and regular security audits to safeguard technical infrastructure. Additionally, the adoption of a Security Information and Event Management system can enhance detection and response capabilities by aggregating and analyzing security data from diverse sources in real time. Conducting periodic security audits remains critical for identifying vulnerabilities and ensuring compliance with evolving cybersecurity standards.

Equally important is cultivating a strong culture of cybersecurity awareness. Employees must understand their responsibilities in protecting organizational assets, including best practices for password management, recognizing phishing attempts, and defending against social engineering and other common attack vectors.

The rise of artificial intelligence (AI) and machine learning (ML) has transformed cybersecurity by enabling automated threat detection, natural language processing for pattern recognition, and real-time response mechanisms. However, these technologies also introduce new risks, particularly adversarial attacks—attempts to manipulate AI models through maliciously crafted inputs. To address this challenge, researchers are developing defensive algorithms capable of identifying such attacks by analyzing anomalies in input data. Parallel efforts focus on designing robust training methodologies that enhance the resilience of AI systems against adversarial manipulation.

### Strengthening Technological Infrastructure

Mitigating the risks associated with DAs is essential in an increasingly interconnected and technology-driven financial environment. The following strategies provide practical guidance for enhancing security, operational resilience, and investor protection:

- Strong security measures: Implement comprehensive security protocols, including MFA, encryption, and intrusion detection systems, to safeguard DAs from unauthorized access and cyber threats.
- Regular software updates: Ensure that all operating systems, applications, and DA management tools are consistently updated to address vulnerabilities and maintain compatibility with evolving security standards.
- Secure storage: Utilize reputable and secure storage solutions (e.g., hardware wallets or institutional-grade custodial services) to minimize exposure to online threats.
- Backup and recovery: Establish a reliable backup and recovery framework by maintaining multiple encrypted copies of DA credentials and access keys in secure, geographically distributed locations.
- Education and awareness: Continuously update knowledge about cybersecurity practices, common threats, and emerging risks within the DA ecosystem to promote informed decision-making.
- Due diligence: Conduct thorough due diligence on DA projects, exchanges, and counterparties to verify credibility, compliance status, and governance practices before investing or transacting.
- Diversification: Spread DA holdings across multiple assets, platforms, and custodial solutions to reduce concentration risk and enhance portfolio resilience.
- Secure communication: Use encrypted and verified communication channels when sharing sensitive DA-related information to prevent interception or identity spoofing.
- Third-party risk assessment: Evaluate the security, regulatory compliance, and operational integrity of third-party service providers or trading platforms before engagement.
- Incident response plan: Develop and routinely test a comprehensive incident response plan that outlines procedures for identifying, containing, and mitigating security breaches or operational disruptions.

### Security protocols and standards

Establishing a strong security culture is the cornerstone of any effective security program. MFA should be implemented to ensure that only authorized users can access organizational resources. Encryption must be employed to protect sensitive data both at rest and in transit, while regular security audits are essential to identify potential vulnerabilities and emerging threats.

Organizations should also consider adopting well-established security standards such as ISO 27001, the NIST Cybersecurity Framework, and Payment Card Industry Data Security Standard (PCI DSS). These standards provide comprehensive frameworks for implementing effective security controls and managing risks within the DA ecosystem.

ISO 27001 is an international standard that outlines best practices for establishing, implementing, maintaining, and continuously improving an Information Security Management System. It specifies requirements for setting security policies and objectives, performing risk assessments and risk management activities, and applying appropriate controls to ensure the confidentiality, integrity, and availability of information.

The NIST Cybersecurity Framework, developed by the U.S. National Institute of Standards and Technology, helps organizations identify, manage, and reduce cybersecurity risks. It provides a common structure and terminology for assessing and communicating cybersecurity risk across internal teams and external stakeholders.

The PCI DSS is a set of technical and operational requirements created by major credit card companies to prevent fraud and protect cardholder data. It establishes clear standards for securing payment card information during storage, processing, and transmission.

Together, these frameworks enable organizations to strengthen their security posture, promote compliance, and enhance resilience against evolving cyber threats in the DA environment.

### Encouraging research in security solutions

In today’s digital landscape, where cyber threats are continuously evolving and becoming increasingly sophisticated, fostering research and development in cybersecurity is of critical importance. For cybersecurity professionals, research is an essential skill that enables them to stay abreast of emerging tools, industry trends, and newly identified vulnerabilities. To advance innovation and strengthen global cyber resilience, various approaches can be taken to support the funding and development of security research initiatives:

- Government funding: Governments can allocate resources to universities, research institutions, and private companies to promote the development of advanced cybersecurity solutions and technologies.
- Public–private partnerships: Collaboration between academia and industry fosters knowledge exchange, interdisciplinary research, and the creation of practical, scalable solutions to real-world cybersecurity challenges.
- Hackathons and competitions: Organizing hackathons, capture-the-flag events, and innovation challenges provides interactive platforms to encourage creativity, problem-solving, and rapid prototyping of security tools.
- Open-source collaboration: Supporting open-source projects encourages transparency, community-driven innovation, and the collective improvement of security solutions.

By combining these approaches, stakeholders can accelerate the pace of discovery, strengthen defenses, and build a more adaptive cybersecurity ecosystem capable of countering emerging digital threats.

Collaboration between the public and private sectors combines the regulatory oversight and policy guidance of government institutions with the innovation, technical expertise, and agility of private enterprises. Public–private partnerships have emerged as a cornerstone of modern cybersecurity strategies, particularly in safeguarding critical infrastructure. Through shared information, coordinated responses, and joint resource allocation, PPPs enhance cyber resilience and reduce vulnerabilities to sophisticated attacks. By leveraging the complementary strengths of both sectors, PPPs promote risk sharing, foster innovation, improve operational efficiency, and generate positive social and economic outcomes.

### Bridging the digital divide

Each time a new DA is introduced, its creators should provide a clear and transparent description of its purpose, structure, and functionality. The literature highlights frequent challenges associated with DAs, including the misuse of private data and insufficient attention to intellectual property protection (Dogru et al. 2018). Nevertheless, distributed data storage can mitigate these concerns by minimizing leakage risks and enhancing overall system security. Even when DAs are designed correctly, their successful adoption depends on a baseline level of financial and economic literacy within society (Ogbonna et al. 2020). Without this foundational understanding, users may struggle to engage meaningfully with DAs or assess their associated risks and benefits.

The emergence of behavioral economics has also influenced attitudes toward consumer protection, which plays a particularly important role in minimizing risks in the DA market. In the case of consumer information problems, traditional economics argues that, wherever possible, the amount of information available to consumers should be increased.

However, in practice, it is unrealistic to expect that, across a wide range of services, consumers will become experts in each field or be as well-informed as professional companies, thereby restoring the equilibrium required for rational decision-making. Yet this expectation is not in the interest of consumers—or society—either. Consumers do not wish to understand in detail how the goods or services they purchase function; rather, they want to use them safely and effectively for their own economic purposes. They would prefer to entrust someone else with the professional task of assessing the relevant conditions and ensuring their fulfillment.

In this way, consumers are relieved of excessive and often ineffective information, freed from unnecessary responsibility, and able to save time. This is particularly true for complex financial services, especially when these are combined with AI applications that are themselves regarded as highly complex, even by experts.

### AI and ML in risk mitigation

Automation, AI, ML, and cloud technologies are becoming essential tools for the financial services industry—particularly within asset management and security services. These technologies promise to usher in a new era of competitive service delivery and efficient processing powered by digital intelligence. However, realizing their full transformational potential requires careful planning and preparation. One of the key challenges lies in proper system training. When the same dataset is used for multiple learning tasks, it often fails to capture the diverse contextual information needed for reliable model performance.

There is growing evidence that credit risk management capabilities can be substantially enhanced through the application of AI and ML techniques, given their ability to semantically interpret unstructured data (Aziz and Dowling 2019). As early as 1994, Altman et al. (1994) conducted a comparative analysis of traditional statistical methods for predicting non-performing and failing loans against a neural network-based alternative, concluding that a combined approach significantly improved predictive accuracy (Machado and Karray 2022).

Consequently, the role of AI and ML in risk mitigation has become increasingly prominent. Several ways in which these technologies can help address risks associated with DAs include:

- Fraud detection: AI and ML can process large volumes of data to identify suspicious patterns indicative of fraudulent activities involving DAs.
- Risk management: By analyzing transaction data in real time, AI and ML models can help financial institutions identify emerging risks and issue timely alerts to enable preventive measures (Jorion 2007; Zihan et al. 2023; Zhang et al. 2017).
- Compliance: Regulatory compliance is central to effective DA risk management. AI and ML systems can assist institutions by automating compliance checks and continuously monitoring transactions for irregularities or potential violations (Tavana et al. 2018; Guerra et al. 2022).
- Cybersecurity: Ensuring the security of DAs is fundamental to safeguarding the broader financial ecosystem. AI and ML tools can help institutions detect potential cybersecurity threats and take proactive steps to avert attacks (Kallel et al. 2024). As the world of CC derivatives evolves at an unprecedented pace, it has been proposed to address emerging challenges by formulating an index that measures market fear using CRIX-based CC options.
- Market analysis: AI and ML can also enhance market analysis by examining large datasets to identify patterns and trends related to DAs. Such analytical capabilities enable financial institutions to make more informed decisions regarding DA investments and risk management (Huang and Lee 2022; Hsu and Lin 2023).

Another important aspect to consider is transparency (i.e., the extent to which the decision-making processes of AI systems can be understood). A lack of interpretability can be particularly problematic in high-stakes contexts such as healthcare, criminal justice, and finance, where incorrect or biased outcomes may have severe consequences. An intuitive approach to developing target-based clustering models that enhance credit scoring transparency is discussed by Teng et al. (2024).

A practical example can be drawn from the use of DA services themselves. When appropriately designed and governed, these services can help mitigate risks in DA management by providing greater security, regulatory compliance, liquidity, convenience, and professional oversight. Nevertheless, before investing in DAs—including NFTs—it remains crucial to select reputable, trustworthy service providers and to carefully evaluate the associated risks.

Among the examples of successful implementations of AI in DA management are the following:

- AI keywording with computer vision: When managing extensive digital libraries, manually tagging every asset can be time-consuming and prone to inconsistency. AI-powered computer vision can automatically analyze images and assign relevant keywords, enabling more efficient organization, retrieval, and categorization of DAs.
- Face recognition in DA management (DAM): AI-based facial recognition allows users to scan images, detect and highlight faces, and assign names or identifiers. Once a face is labeled, the DA management system can automatically apply that metadata to all matching instances across the library. This functionality enables users to search and organize content using facial metadata, significantly improving efficiency. The key advantage of AI-driven auto-tagging in DAM systems lies in its ability to apply metadata to millions of assets quickly and without human intervention. This innovation removes one of the major bottlenecks in DA ingestion—manual metadata tagging—and enhances the scalability and accuracy of asset management workflows.

AI and ML offer numerous advantages for DAM, including automated tagging, improved searchability, reduced manual labor and human error, optimized workflows, and enhanced security and compliance. In the future, AI-driven analytics and generative models may further expand the capabilities of DAM systems. Additionally, the integration of AI and ML can help mitigate risks associated with the rapid proliferation of DAs by analyzing large volumes of data in real time, detecting anomalies, preventing fraud, ensuring regulatory compliance, strengthening cybersecurity, and providing valuable insights into market trends.

An example of applying AI and ML to mitigate DA-related risks is provided by Pele et al. (2021), who employed ML techniques to demonstrate that the primary factor distinguishing cryptocurrencies from traditional assets is the tail factor. Based on this finding, it becomes possible to assess the impact of including cryptocurrencies in a traditional asset portfolio from the perspective of market risk measures such as VaR, volatility, and the Sharpe Ratio.

**Fig. 8:**
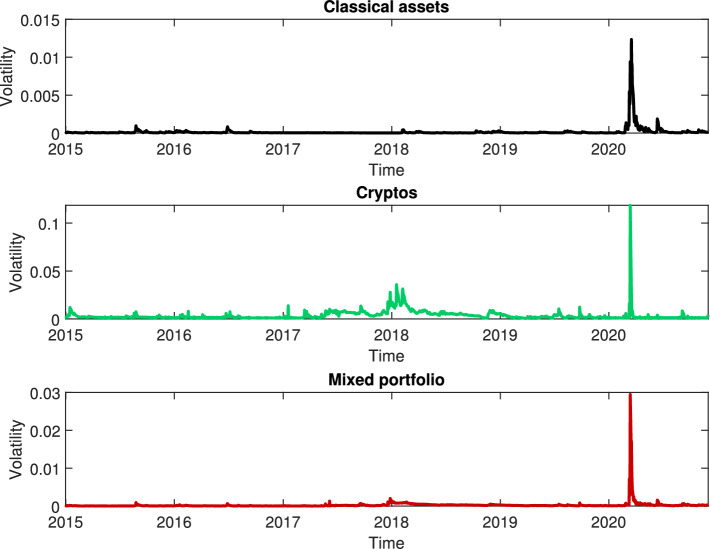
Estimated volatilities from Student’s t GJR-GARCH(1,1,1) model https://quantinar.com/course/87/classify_cryptos

Incorporating cryptocurrencies into a traditional asset portfolio tends to increase both volatility and VaR. During the COVID-19 pandemic, for instance, the 1% VaR rose from 8% for a portfolio composed solely of traditional assets to 11% for a mixed portfolio that included cryptocurrencies. Figure 8 illustrates this effect, showing volatility estimates derived from a Student’s t GJR-GARCH(1,1,1) model across different portfolio configurations. The results indicate that the CC portfolio exhibits peak volatility nearly ten times higher than that of traditional assets, while the mixed portfolio’s volatility peaks at roughly three times that of the classical asset portfolio.

In conclusion, the growing presence of DAs introduces new forms of risk into the global financial ecosystem. However, AI and ML offer powerful tools for mitigating these risks. By analyzing vast datasets in real time, these technologies can detect potential threats, prevent fraud, ensure regulatory compliance, strengthen cybersecurity, and generate actionable insights into evolving market dynamics. As DAs continue to expand in scope and adoption, AI and ML will play an increasingly central role in managing and reducing associated risks.

## Toward a resilient and inclusive DA ecosystem

The exploration of DAs in the emerging digital era is nothing short of transformative. In this position paper, we have defined DAs, examined their multifaceted risk profiles, and proposed proactive strategies for mitigation. As the understanding of DAs deepens and their applications expand, it becomes increasingly vital to address these risks through forward-looking approaches. The roadmap outlined here aims to guide the development of a resilient, transparent, and inclusive DA ecosystem.

It is evident that the digital realm is evolving at a pace that surpasses the capacity of traditional financial and regulatory frameworks. From the early conception of digital currencies to the emergence of complex instruments such as NFTs, CBDCs, and DeFi protocols, the landscape of DAs has expanded considerably. Each type of DA introduces distinct challenges and opportunities. The foundational blockchain technology underlying many DAs has fundamentally redefined notions of trust, payment, and value exchange.

Risks, particularly those accompanying technological innovation, are inevitable. History demonstrates that every major technological advance brings vulnerabilities alongside progress. Yet while technological challenges are significant, they are often identifiable and amenable to research-driven solutions. Socioeconomic risks, by contrast, highlight a deeper concern: although DAs hold the promise of democratization, they can also reinforce or exacerbate existing inequalities. Concerns over regime shifts and unequal access to digital financial services underscore the persistence of the digital divide. Finally, the environmental footprint of BC cryptocurrencies remains a pressing issue. As global efforts intensify to combat climate change, the DA ecosystem must critically assess its practices and innovate toward a more sustainable and responsible future.

Mitigating these risks is not a linear process but a multifaceted endeavor requiring coordinated action across technical, institutional, and societal dimensions. Strengthening technological infrastructures must be accompanied by the establishment of rigorous security protocols and governance standards. Designing resilient systems is only the first step; continuous research and innovation in cybersecurity and risk management remain essential to address evolving threats.

Bridging the digital divide, though a broader social objective, carries particular urgency within the DA ecosystem. Inclusivity must be embedded into design and policy from the outset to ensure that DAs do not become a privilege of the technologically or economically advantaged, but an accessible opportunity for all. Equally, the potential of AI and ML in identifying, assessing, and mitigating risks offers a promising pathway toward smarter, more adaptive oversight.

Ultimately, the path toward a resilient and inclusive DA ecosystem depends on collaboration. Stakeholders (i.e., technologists, regulators, investors, and end users) must engage in sustained dialogue and coordinated action. While DAs are inherently decentralized, their safe and equitable integration into global markets requires a shared framework of collective intelligence, accountability, and cooperation.

In the current policy landscape—illustrated by ongoing discussions such as former and elected President Trump’s debate on cryptocurrencies—we observe a generally positive trajectory toward the development and institutionalization of DA ecosystems. Any emerging forms of DAs, whether NFTs, energy tokens, or other variants, will ultimately need to conform to the regulatory principles outlined in Sect. 2. In Sect. 3, we examined in detail the risk profiles of various DAs, including NFTs. The regulatory framework presented herein seeks to comprehensively address these challenges, offering a structured path for regulators and investors to effectively manage the risks inherent in the evolving digital asset ecosystem.

Nevertheless, policymakers face the complex task of understanding, analyzing, and regulating this rapidly transforming space. On one hand, DAs hold significant promise for mitigating traditional financial-sector risks, enhancing financial inclusion, and promoting transparent, verifiable, and fair financial infrastructures. On the other hand, mounting evidence indicates that many projects operating under the DA label are, in practice, less decentralized than their rhetoric suggests.

Governance within the smart contract environment determines how privileged functions are executed and is typically organized under two principal architectures: account-based and token-based governance. Each model offers distinct advantages but also introduces varying degrees of centralization risk, underscoring the importance of careful design and oversight in maintaining the integrity of decentralized systems.

In operational terms, the governance of many DeFi protocols is often administered through one or more externally owned accounts endowed with privileged access—commonly referred to as *admin keys*. This design offers clear advantages in terms of efficiency and responsiveness, allowing for the swift implementation of upgrades, emergency shutdowns, and parameter adjustments. However, such centralized control introduces inherent vulnerabilities that stand in tension with the principles of decentralization these systems espouse.

Transparency regarding the identity of admin key holders can enhance public trust and accountability, but it also creates new attack vectors. When key holders are publicly identifiable, they may become targets of coercion, extortion, or blackmail—risks that are particularly acute in jurisdictions lacking robust legal protections or where malicious actors are active. Conversely, maintaining full anonymity can impede accountability and raise concerns about governance legitimacy, insider capture, or untraceable decision-making.

Several real-world cases illustrate the material risks associated with admin key centralization. In the case of the **bZx protocol**, attackers exploited private keys obtained through a phishing attack on a developer, resulting in cumulative losses exceeding USD 8 M across multiple incidents between 2020 and 2021 (Qin et al. 2021). This episode underscored the dangers of concentrated administrative authority and insufficient key management practices. Similarly, the **Harvest Finance** exploit of October 2020—during which approximately USD 24 M in stablecoins were rapidly withdrawn—exposed vulnerabilities related to insider access and opaque governance structures (Vidal-Tomás et al. 2023). Although insider involvement was never conclusively established, the exploit demonstrated how centralized administrative privileges can enable rapid depletion of funds with limited oversight or safeguards.

To mitigate these vulnerabilities, several DeFi protocols have introduced or proposed hybrid governance structures. In these models, administrative authority is distributed among multiple key holders, typically comprising a mix of anonymous and publicly identifiable entities. This design seeks to balance competing concerns: anonymity helps protect individuals from coercion or physical threats, while identifiable stakeholders serve as visible points of accountability and trust. Complementary technical safeguards (e.g., multi-signature arrangements and timelock mechanisms) further minimize single points of failure, enabling community members to review and challenge governance actions before execution.

Beyond hybrid key management, evolving decentralized autonomous organization governance frameworks present additional avenues for mitigating centralization risk. Mechanisms such as time-delayed execution, community review periods, and layered quorum thresholds can enhance transparency and procedural fairness, while constraining unilateral decision-making. Nonetheless, these measures must be grounded in robust threat modeling and a nuanced understanding of social dynamics, particularly the risks of voter apathy, token concentration, and governance capture.

Token-based governance links voting power directly to governance token holdings, reflecting the assumption that participants with greater financial stakes should exert proportionally more influence. In some implementations, voting is non-binding—token holders may propose or endorse changes, but final authority rests with administrators controlling key permissions. In more decentralized frameworks, binding votes automatically enact approved proposals once quorum or threshold conditions are satisfied, thereby enhancing procedural legitimacy and reducing reliance on trusted intermediaries.

A persistent challenge, however, lies in ownership concentration. When a small number of entities accumulate substantial token holdings, decision-making risks becoming effectively centralized. Moreover, anonymity of wallet addresses and the use of token-wrapping or staking derivatives can obscure actual control structures, complicating transparency and accountability efforts (Schuler et al. 2024).

In conclusion, the journey of DAs is still in its early stages. The path forward is rich with both promise and complexity. By recognizing the multifaceted challenges, understanding their structural nuances, and applying innovative, well-calibrated mitigation strategies, stakeholders can help ensure that DAs fulfill their transformative potential. Ultimately, the future of DAs depends not only on technological advancement but also on the creation of a secure, inclusive, and sustainable ecosystem—one capable of delivering shared value and enduring trust.

## Appendix

List of abbreviations and descriptions.

**Table:**

Abbreviation	Description
5AMLD	The Fifth Anti-Money Laundering Directive
AI	Artificial Intelligence
AML	Anti-Money Laundering
API	Application Programming Interfaces
ARTs	Asset-Referenced Tokens
BC	Blockchain
BCBS	Basel Committee on Banking Supervision
BIS	Bank for International Settlements
BPMN	Business Process Modeling Notation standard version 2.0
BTC	Bitcoin
CBDC	Central Bank Digital Currency
CC	Cryptocurrency
DA	Digital Asset
DAI	Digital Art Index
DeFi	Decentralized Finance
DEX	Decentralized Exchange
DFP	Digital Finance Package
DLT	Distributed Ledger Technology
DORA	Digital Operational Resilience Act
EBA	European Banking Authority
ECB	European Central Bank
EMTs	Electronic Money Tokens
ERP	Enterprise Resource-Planning System
ESMA	European Securities and Markets Authority
ETH	Ethereum
FRM	Financial Risk Meter
FSB	Financial Stability Board
HSM	Hardware security module
ICO	Initial Coin Offering
ICT	Information and communications technology
IS	Information System
ISP	Internet Service Provider
IT	Information Technology
IT/IS	Information Technology and Information System
KYC	Know Your Customer
LRD	Liberty Reserve Dollar
MEV	Maximal Extractable Value
MFA	Multi-Factor Authentication
MiCA	Markets in Crypto-Assets
ML	Machine Learning
NFT	Non-Fungible Token
NLP	Natural Language Processing
PII	Personal Identifying Information
QE	Quantitative Easing
SEC	Securities and Exchange Commission
SIEM	Security Information and Event Management
VASP	virtual asset service provider
VIX	Volatility Uncertainty Index
WAF	Web Application Firewall
XAI	Explainable Artificial Intelligence
